# Genotypic and phenotypic landscapes of 51 pharmacogenes derived from whole-genome sequencing in a Thai population

**DOI:** 10.1371/journal.pone.0263621

**Published:** 2022-02-17

**Authors:** Natnicha Wankaew, Pajaree Chariyavilaskul, Monpat Chamnanphon, Adjima Assawapitaksakul, Wanna Chetruengchai, Monnat Pongpanich, Vorasuk Shotelersuk

**Affiliations:** 1 Program in Bioinformatics and Computational Biology, Graduate School, Chulalongkorn University, Bangkok, Thailand; 2 Clinical Pharmacokinetics and Pharmacogenomics Research Unit, Faculty of Medicine, Chulalongkorn University, Bangkok, Thailand; 3 Department of Pharmacology, Faculty of Medicine, Chulalongkorn University, Bangkok, Thailand; 4 Department of Pathology, Faculty of Medicine, Srinakharinwirot University, Nakornnayok, Thailand; 5 Center of Excellence for Medical Genomics, Medical Genomics Cluster, Department of Pediatrics, Faculty of Medicine, Chulalongkorn University, Bangkok, Thailand; 6 Excellence Center for Genomics and Precision Medicine, King Chulalongkorn Memorial Hospital, the Thai Red Cross Society, Bangkok, Thailand; 7 Department of Mathematics and Computer Science, Faculty of Science, Chulalongkorn University, Bangkok, Thailand; 8 Age-related Inflammation and Degeneration Research Unit, Chulalongkorn University, Bangkok, Thailand; Jordan University of Science and Technology, JORDAN

## Abstract

Differences in drug responses in individuals are partly due to genetic variations in pharmacogenes, which differ among populations. Here, genome sequencing of 171 unrelated Thai individuals from all regions of Thailand was used to call star alleles of 51 pharmacogenes by Stargazer, determine allele and genotype frequencies, predict phenotype and compare high-impact variant frequencies between Thai and other populations. Three control genes, *EGFR*, *VDR*, and *RYR1*, were used, giving consistent results. Every individual had at least three genes with variant or altered phenotype. Forty of the 51 pharmacogenes had at least one individual with variant or altered phenotype. Moreover, thirteen genes had at least 25% of individuals with variant or altered phenotype including *SLCO1B3* (97.08%), *CYP3A5* (88.3%), *CYP2C19* (60.82%), *CYP2A6* (60.2%), *SULT1A1* (56.14%), *G6PD* (54.39%), *CYP4B1* (50.00%), *CYP2D6* (48.65%), *CYP2F1* (46.41%), *NAT2* (40.35%), *SLCO2B1* (28.95%), *UGT1A1* (28.07%), and *SLCO1B1* (26.79%). Allele frequencies of high impact variants from our samples were most similar to East Asian. Remarkably, we identified twenty predicted high impact variants which have not previously been reported. Our results provide information that contributes to the implementation of pharmacogenetic testing in Thailand and other Southeast Asian countries, bringing a step closer to personalized medicine.

## Introduction

Genetic variations play an important role in personalized drug response and constitute pharmacogenetics biomarkers for drug dosing, effectiveness, and toxicity [1]. Variant frequencies in pharmacogenes differ significantly among various populations [2–4]. However, these data in Asian populations, especially Thai, are limited. A meta-analysis of 56,945 individuals studying allele frequencies of 12 *CYP* genes in several ethnicities, but only 7.6% were data of East Asians [2]. A study in the United States provides allele frequencies in *CYP2D6* of 104,509 individuals where only 0.2% were Asians [5].

High-throughput DNA sequencing technologies with decreasing costs lead genome and exome sequencing to be increasingly used in clinical medicine. They provide the molecular diagnosis for the primary disease and offer a determination of genetic variations in pharmacogenes. To the best of our knowledge, there has been only one study in the Thai population using genome sequencing to investigate pharmacogenes [6]. The participants, however, were affected with Brugada syndrome, which is more common in the Northeastern part of Thailand; therefore, the results may not represent the general Thai population. In addition, the study determined variants in only 25 pharmacogenes. Variants of other 26 genes associated with the pharmacokinetics of drugs treating the top 10 diseases causing the highest mortality for Thais (https://www.cdc.gov/globalhealth/countries/thailand/) including cancer, non-communicable diseases, and infections have not been determined.

In an effort to determine the star allele profile for a more comprehensive list of relevant and meaningful pharmacogenes in a general Thai population, this study explored allele frequencies, genotype frequencies, phenotype prediction together with deleterious variants in 51 pharmacogenes using WGS data of 171 unrelated healthy Thai individuals from all geographical regions of Thailand.

## Materials and methods

### Study participants

The genome sequencing data of 171 healthy unrelated Thais parents of children with various rare diseases visiting the Genetics Clinic of King Chulalongkorn Memorial Hospital were enrolled in the study.

### Ethics statement

The study was approved by the Institutional Review Board of the Faculty of Medicine, Chulalongkorn University (IRB number 264/62). All participants provided their written informed consent.

### The margin of error calculation

The margin of error was calculated based on Cochran’s sample size formula [7] at a 95% confidence interval, with a sample size of 171, the Thai population size of approximately 67 million people, and a population proportion having an attribute, e.g., normal phenotype at 0.5, which would yield a maximum margin of error.

### Star allele analysis

We first assessed the quality of our data using FastQC (http://www.bioinformatics.babraham.ac.uk/projects/fastqc). Next, we used BWA [8] version 0.7.17 (BWA-MEM algorithm) to align reads in Fastq files to the reference genome hg19. To speed up the computation, we extracted reads from genome location of 51 pharmacogenes including:- *CACNA1S*, *CFTR*, *CYP1A1*, *CYP1A2*, *CYP1B1*, *CYP2A6*, *CYP2A13*, *CYP2B6*, *CYP2C8*, *CYP2C9*, *CYP2C19*, *CYP2D6*, *CYP2E1*, *CYP2F1*, *CYP2J2*, *CYP2R1*, *CYP2S1*, *CYP2W1*, *CYP3A4*, *CYP3A5*, *CYP3A7*, *CYP3A43*, *CYP4B1*, *CYP26A1*, *CYP4F2*, *CYP19A1*, *DPYD*, *G6PD*, *GSTM1*, *GSTP1*, *GSTT1*, *IFNL3*, *NAT1*, *NAT2*, *NUDT15*, *POR*, *RYR1*, *SLC15A2*, *SLC22A2*, *SLCO1B1*, *SLCO1B3*, *SLCO2B1*, *SULT1A1*, *TBXAS1*, *TPMT*, *UGT1A1*, *UGT1A4*, *UGT2B7*, *UGT2B15*, *UGT2B17*, and *VKORC1*.

Once the bam files were obtained, we used GATK version 4.1.3.0 [9, 10] and followed GATK best practice workflow to obtain called variants in a variant call format (VCF) file. Namely, we identified duplicate reads with the MarkDuplicates tool then performed base quality score recalibration (BQSR). To call variants, HaplotypeCaller per-sample, which generated GVCF file for each sample, was applied, then all GVCFs files were combined, and joint calling using GenotypeGVCFs was performed. Then, we filtered variants with the following criteria. Single nucleotide variants (SNVs) that did not match any of these conditions—QualByDepth (QD) <2.0, FisherStrand (FS) >60.0, RMSMappingQuality (MQ) <40, MappingQualityRankSumTest (MQRankSum) <12.5, and ReadPosRankSumTest (ReadPosRankSum) <-8.0—were passed. Indels that did not match any of these conditions—QD <2.0, FS >200.0, and ReadPosRankSum <-20.0—were passed.

Afterwards, we obtained read depth in the region of all 51 genes for all samples using GATK-DepthOFCoverage (version 3.8.1) which calculated depth from bam files. For compatibility with Stargazer (https://stargazer.gs.washington.edu/stargazerweb), we referred to this read depth file as a target GDF (GATK-DepthOfCoverage Format) file.

To call star alleles and obtain predicted phenotypes, we used Stargazer [11, 12] version 1.0.8, where *EGFR*, *VDR*, and *RYR1* were used as a control gene one at a time. Inputs that Stargazer requires are VCF and GDF files, which were obtained as described previously. Stargazer internally used Beagles [13, 14] to phase haplotype with the 1000 genomes project (1KGP) as a reference panel. The reference panel was changed to only East and Southeast Asian samples.

### Pathogenic pharmacogenetic variants analysis

The VCF file was used to calculate allele frequency by plink (version 1.7). Single nucleotide variants and small insertion and deletions were identified as known pharmacogenetic variants by available information from dbSNP (https://www.ncbi.nlm.nih.gov/snp) and PharmGKB (https://www.pharmgkb.org) databases. All variants were annotated with 1000 genomes global and continental minor allele frequencies and variant consequences and its impact on protein function using Ensemble Variant Effect Predictor web tools (http://grch37.ensembl.org/Homosapiens/Tools).

The distribution of high impact variants was compared among Thais (THA) and the five populations from 1000 genomes project (https://www.internationalgenome.org/data), which are Africans (AFR), Americans (AMR), East Asians (EAS), European (EUR), and South Asians (SAS) using Chi-square test. The statistical analyzes were completed by the standard statistical package (R version 4.0.3), and a p-value of <0.01 was used as a significant level after controlling for the critical false discovery rate of 0.05 by the Benjamini-Hochberg procedure [15].

## Results

### Population

Data from 171 individuals (male/female = 91/80) were analyzed. Geographical areas were available from 65.5% of participants and showed that they were from all regions of Thailand (S1 Table in S2 File). With 171 individuals, the maximum margin of error for the proportion of population carrying certain star alleles is approximately 7.5% at a 95% confidence level.

### Sequence quality and read depth

**Fig 1:**
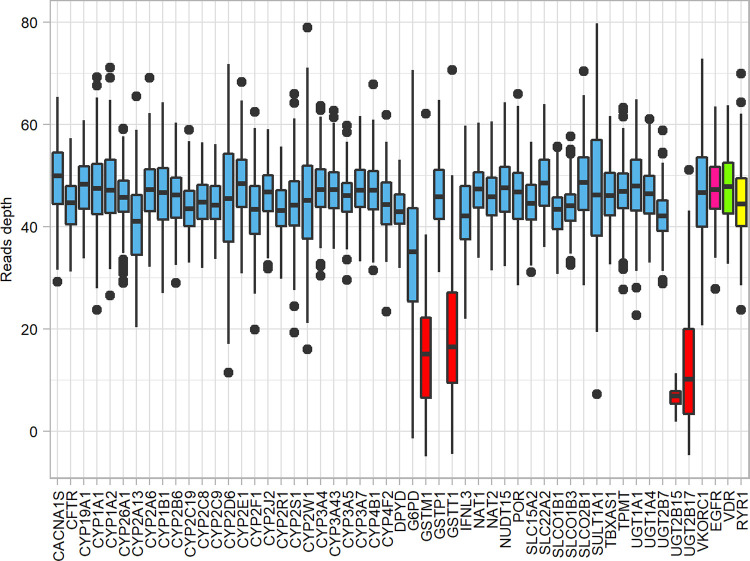
Average read depth. The overview of the average read depths of the 51 pharmacogenes (blue and red) and three control genes, *EGFR* (pink), *VDR* (green), and *RYR1* (yellow). Four red boxes represent genes with low depth coverage, which are *GSTM1*, *GSTT1*, *UGT2B15*, and *UGT2B17*.

Quality scores across all bases of all samples were >30. All genes except *G6PD*, *GSTM1*, *GSTT1*, *UGT2B15*, and *UGT2B17* had an average read depth >40x (Fig 1). Among three control genes, *EGFR* had the highest average read depth with a mean ± standard deviation (SD) of 43.91 ± 7.45 followed by *VDR* (43.30 ± 7.76) and *RYR1* (40.39 ± 7.82).

### Control genes

The three available control genes in Stargazer yielded 99.08% identical results where *EGFR vs VDR*, *EGFR vs RYR1*, and *VDR vs RYR1* had 99.75%, 99.09%, and 99.31% identical results, respectively.

Using *EGFR* as the control gene would be unable to predict genotypes of two genes, *UGT2B15* (n = 99) and *CYP2D6* (n = 7), followed by *VDR* with three genes, *UGT2B15* (n = 92), *CYP2D6* (n = 5), and *SLC22A2* (n = 1), and *RYR1* with four genes, *UGT2B15* (n = 83), *CYP2D6* (n = 8), *SLC22A2* (n = 2), and *CYP2E1* (n = 1) (S1A Fig in S1 File).

*UGT2B15* showed the highest incidence of unpredictable genotype, followed by *CYP2D6*, *SLC22A2*, and *CYP2E1*. Stargazer was able to call star alleles in 164, 165, and 162 individuals using *EGFR*, *VDR*, and *RYR1* as the control genes, respectively (S1B Fig in S1 File). The results presented here were based on *EGFR* as the control gene.

### Allele and genotype frequencies and phenotype prediction

**Fig 2:**
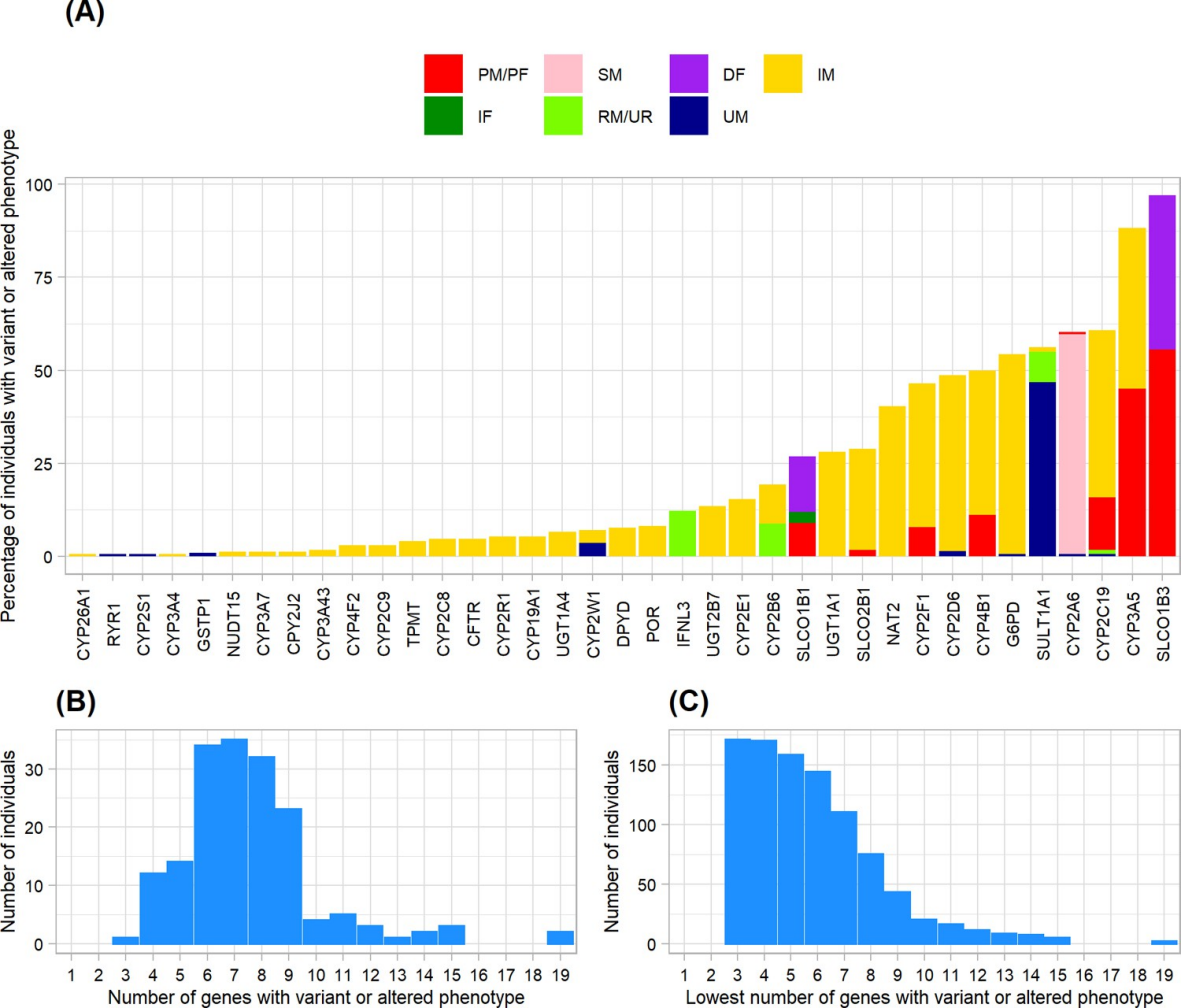
Variant or altered phenotype: Genes and number of individuals. (A) Percentage of individuals with variant or altered phenotype. Poor metabolizer (PM; red) or poor function (PF; red) for *SLCO1B1*, *SLCO1B3*, and *SLCO2B1*; slow metabolizer (SM; pink); decreased function (DF; purple); intermediate metabolizer (IM; yellow); increased function (IF; dark green); rapid metabolizer (RM; green) or unfavorable response (UR; green) for *IFNL3*; ultra-rapid metabolizer (UM; dark blue). (B) The number of individuals with a particular number of genes where individuals had variant or altered phenotype. (C) The number of individuals with at least a particular number of genes where individuals had variant or altered phenotype.

The thirteen genes that more than 25% of our samples had variant or altered phenotype were *SLCO1B3*, *CYP3A5*, *CYP2C19*, *CYP2A6*, *SULT1A1*, *G6PD*, *CYP4B1*, *CYP2D6*, *CYP2F1*, *NAT2*, *SLCO2B1*, *UGT1A1*, *SLCO1B1* (Fig 2A, S2-S5 Figs in S1 File). Overall, Stargazer detected 196 star alleles in 51 genes (S2 Table in S2 File). Stargazer was unable to call star allele for some genes or predict phenotype (unknown phenotype) in specific individuals. Therefore, the percentage calculated in Fig 2A was based on the number of informative phenotypes, i.e., excluding individuals with uncalled and unknown phenotypes.

Only 5 individuals (2.9%) had SLCO1B3*1/*1 normal function phenotype. SLCO1B3*S1, a decreased-function allele, is the most common allele present in 166 individuals (97.1%). Of these, 95 individuals (55.6%) had poor-function phenotype (SLCO1B3*S1/*S1 or SLCO1B3*S1/*DEL) and 71 individuals (41.5%) had decreased-function phenotype (SLCO1B3*1/*S1) (Fig 2A, S4 Fig in S1 File).

CYP3A5*3, a no-function allele, was observed in 74 individuals (43.3%) with the intermediate metabolizer phenotype (CYP3A5*1/*3) (S2C Fig in S1 File) and 77 individuals (45.0%) with the poor metabolizer phenotype (CYP3A5*3/*3) (Fig 2A, S2 Fig in S1 File).

In *CYP2C19*, 77 individuals (45.0%) were classified as intermediate metabolizers carrying a gene deletion allele (*DEL) or one no-function *2, *3, or *5 allele (CYP2C19*1/*2, *1/*3, *1/*5, and *1/*DEL). 24 individuals (14%) were predicted to be poor metabolizers carrying two no-function *2 and *3 alleles (CYP2C19*2/*2, *2/*3, and *3/*3). Increased-function CYP2C19*17 allele was found in two individuals with the rapid metabolizer phenotype (CYP2C19*1/*17) and one individual with the ultra-rapid metabolizer phenotype (CYP2C19*17*/17) (Fig 2A, S2 Fig in S1 File).

In *CYP2A6*, 98 individuals (59%) were predicted to be slow metabolizers carrying one or more decreased-function alleles (CYP2A6*7, *9, *10, *11, *12, *19, *21, and *35) or no-function allele (CYP2A6*4). Diplotypes were CYP2A6*1/*4, *1/*7, *1/*9, *1/*10, *1/*12, *1/*19, *1/*35, *4/*7, *4/*9, *4/*11, *4/*35, *7/*12, *7/*21, *10/*11, and *10/*35. One individual was classified as poor metabolizer (CYP2A6*4/*4) and one was ultra-rapid metabolizer (CYP2A6*1/*1x2) (Fig 2A, S2 Fig in S1 File).

In *SULT1A1*, several diplotypes carrying ≥2 copies of a gene (SULT1A1*1/*1x2, *1/*1x3, *1/*1x4, *1/*1x5, *1x2/*1x2, *1x2/*1x3, *1x2/*1x6, *1x2/*2x2, *1x2/*2x3, *1x3/*1x6, and *1x3/*2x3) were predicted as ultra-rapid metabolizers (n = 80; 46.8%). Fourteen individuals (8.2%) carried SULT1A1*1x2/*2 diplotypes and were predicted to be rapid metabolizers. In addition, two individuals predicted to be intermediate metabolizers (SULT1A1*1/*S1 and SULT1A1*2/*2) as they carried SULT1A1*S1 no-function allele and SULT1A1*2 decreased-function allele (Fig 2A, S3 Fig in S1 File).

The allele frequencies of G6PD*1 (normal-function) and G6PD*DEL (no-function) alleles were the two most common *G6PD* alleles occurring at 66.1% and 26.6%, respectively. G6PD*DEL allele along with other deficiency alleles (G6PD*8, *21, *28, *31, *50, and *51) comprised diplotypes (G6PD*1/*DEL, *21/*DEL, *28/*DEL, *51/*51, *51/*DEL, and *8/*DEL) which had predicted phenotype as intermediate metabolizer in 92 individuals (53.8%). Out of 14 diplotypes, G6PD*1/*DEL had the highest frequency (49.1%) followed by G6PD*1/*1 (36.8%). In addition, one individual had G6PD*1/*1x2 diplotype and was predicted to be an ultra-rapid metabolizer. A high frequency of a deletion observed corresponded to male subjects (Fig 2A, S5 Fig in S1 File).

Thirty-five individuals (38.9%) carrying CYP4B1*1/*2 and ten individuals (11.1%) carrying CYP4B1*2/*2 or CYP4B1*S1/*2 were predicted to be intermediate and poor metabolizers, respectively (Fig 2A, S2 Fig in S1 File).

Nineteen distinct *CYP2D6* alleles were detected in our subjects (S2D Fig in S1 File). These included gene duplication (CYP2D6*1x2, *2x2, *2x3, *10x2, and *39x2), gene deletion (CYP2D6*5) and gene rearrangement (CYP2D6*36+*10 and *36x3+*10). CYP2D6*36+*10 and CYP2D6*10 decreased-function alleles were the most common allele found and contributed to predicted intermediate metabolizer phenotype (CYP2D6*10/*10, *10/*36+*10, *10/*36x3+*10, *10/*41, *36+*10/*36+*10, *36+*10/*41, *4/*10, *4/*36+*10, *5/*10, and *5/*36+*10) in 62 individuals. Moreover, intermediate metabolizer phenotype (CYP2D6*1/*4, *1/*5, *2/*5, *4/*41, *5/*39, and *5/*41) was resulted from other decreased-function (CYP2D6*41) and no-function alleles (CYP2D6*4 and CYP2D6*5) in 7 individuals. In addition, two subjects were classified as ultra-rapid metabolizer (CYP2D6*1/*2x2 and CYP2D6*1/*2x3) (Fig 2A, S2 Fig in S1 File).

CYP2F1*2 (no-function allele) contributed to intermediate metabolizer phenotype (CYP2F1*1/*2) in 59 individuals (38.6%) and poor metabolizer phenotype (CYP2F1*2/*2) in 12 individuals (7.8%) (Fig 2A, S2 Fig in S1 File).

NAT2*5, *6, *7 decreased-function alleles and NAT2*DEL no-function allele comprised intermediate metabolizer diplotypes (NAT2*1/*DEL, *5/*6, *5/*7, *5/*DEL, *6/*6, *6/*7, *6/*DEL, *7/*7, and *7/*DEL) in 69 individuals (40.4%) (Fig 2A, S3 Fig in S1 File).

A large number of the subjects (71.1%) had SLCO2B1*1/*1 normal function phenotype. However, 57 individuals had unknown phenotype (SLCO2B1*1/*S464F, *S1/*S464F, and *S464F/*S464F) due to the unknown function of *S464F allele. A no-function SLCO2B1*S1 allele contributed to intermediate function phenotype (SLCO2B1*1/*S1) in 31 individuals (27.2%) and poor-function phenotype (SLCO2B1*S1/*S1) in 2 individuals (1.8%) (Fig 2A, S4 Fig in S1 File).

UGT1A1*6, *7, *27, and *60 decreased-function alleles comprised intermediate metabolizer diplotypes (UGT1A1*27/*27, *27/*60, *6/*27, *6/*6, *6/*60, *6/*7, and *60/*60) in 48 individuals (28%) (Fig 2A, S3 Fig in S1 File).

Poor-, decreased-, normal- and increased-function phenotypes were observed for *SLCO1B1*. SLCO1B1*15 and SLCO1B1*17 decreased-function allele and SLCO1B1*DEL no-function allele comprised diplotypes (SLCO1B1*15/*17, *15/*DEL, *17/*DEL, and *1B/*DEL) that were predicted to be poor-function phenotype in 15 individuals (8.9%). Twenty-five individuals (14.3%) had decreased-function phenotype carrying SLCO1B1*1/*15, *1/*17, *1B/*15, *1B/*17, or *35/*DEL where *1B was a normal-function allele and *35 was an increased-function allele. Five individuals (3%) carrying SLCO1B1*1/*35, *14/*35, *1B/*35 were predicted with increased-function phenotype where *14 was an increased-function allele (Fig 2A, S4 Fig in S1 File).

A high frequency of a no-function allele (deletion) was observed in *GSTM1*, *GSTT1*, *UGT2B17*, and *UGT2B15* and this corresponded with poor or intermediate metabolizer phenotype in the first three genes and unknown or unpredictable phenotype in *UGT2B15* (S3 Fig in S1 File). Nevertheless, results in these four genes should be interpreted with caution due to a very low read depth, which might explain a high frequency of a deletion observed (S3 Fig in S1 File).

All individuals had normal phenotype for *CACNA1S* and had unknown phenotypes for *VKROC1*. In *CYP1A1*, *CYP1A2*, *CYP1B1*, *CYP2A13*, *NAT1*, *SLC15A2*, *SLC22A2*, and *TBXAS1*, the predicted phenotypes were either normal metabolizers or unknown.

Individuals mostly have variant or altered phenotypes in 6–8 genes (Fig 2B). Every individual has variant or altered phenotype in at least three genes but no more than 19 genes. One hundred and fifty-eight individuals had variant or altered phenotypes in at least five genes (Fig 2C).

### Pharmacogenetic variants frequencies comparison

**Fig 3:**
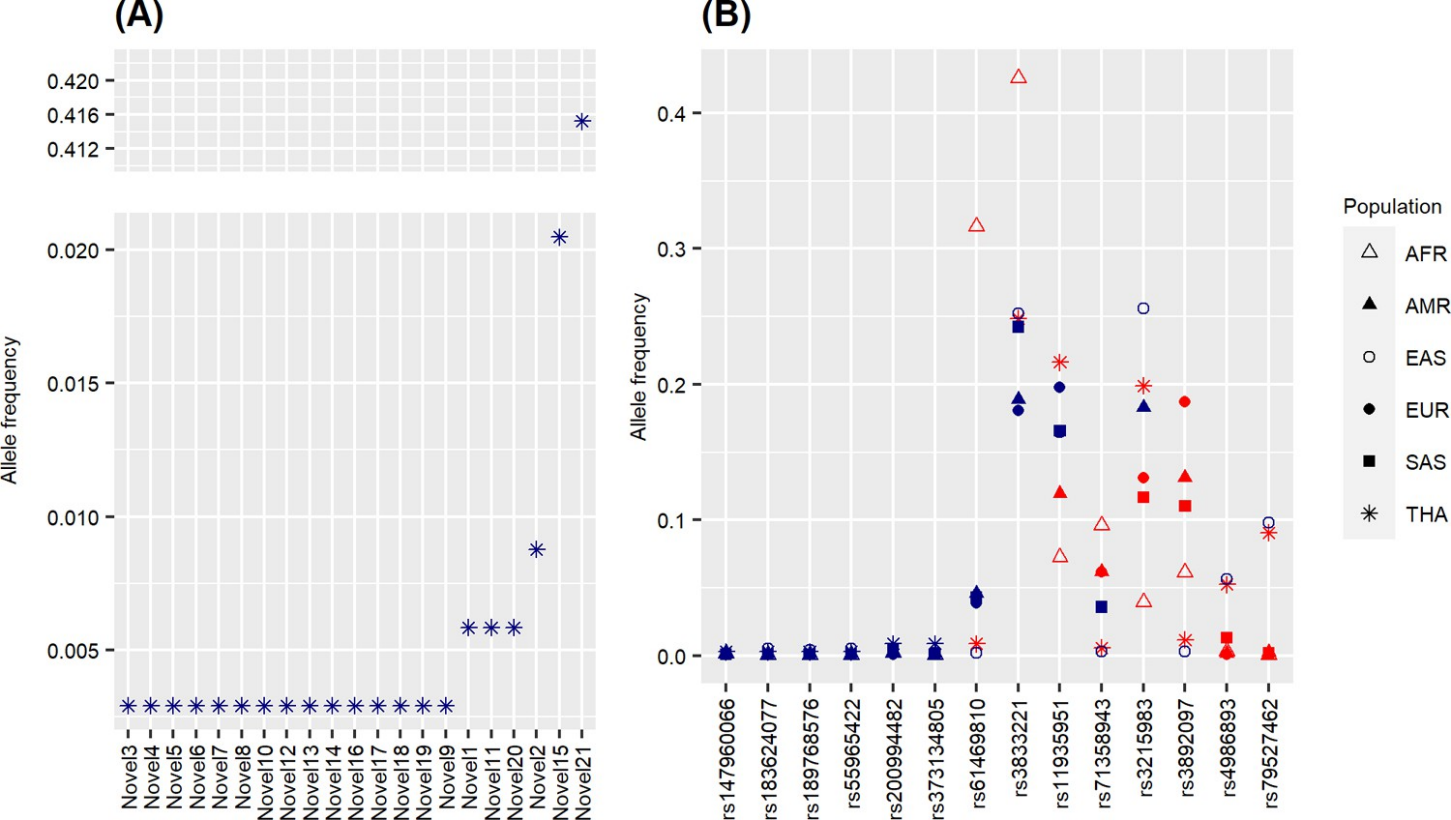
Allele frequency comparisons of the 35 high impact variants among Thais (THA), African (AFR), admixed American (AMR), East Asian (EAS), European (EUR), and South Asian (SAS) were obtained from the 1000 genome project database. (A) Variants whose allele frequencies are not available on the 1000 Genome Project database but found in Thai are denoted with Novel1-Novel21. (B) Δ, ▲, ○, ●, ■, * denote allele frequency of AFR, AMR, EAS, EUR, SAS, and THA, respectively. Allele frequencies that were significantly different (p < 0.05) between THA and other populations were shown in red, and the non-statistically significant variants were shown in blue.

We identified 28,061 variants within 51 pharmacogenes in the 171 individuals, where the majority were intron variants (S3 Table in S2 File). Variants were classified according to their functional consequences, for example, frameshift, missense or synonymous. There were 322 novel variants with high, moderate or low impacts (S3 Table in S2 File). Of these, 21 (Novel1 to Novel21) were high-impact variants, which had not been reported in 1000 Genomes Project Phase 3 (Table 1; Fig 3). However, one of them, Novel6, which corresponds to rs370320936, has been reported in Singaporean and Malaysian (https://www.ncbi.nlm.nih.gov/projects/SNP/snp_retrieve.cgi?subsnp_id=657184749). Therefore, twenty variants were novel. Allele frequencies of 6 variants representing *DPYD* (rs189768576), *CYP3A5* (rs373134805, rs55965422), *SLCO1B1* (rs200994482, rs183624077), and *CYP2D6* (rs147960066) were not significantly different among the six populations (Fig 3B). The remaining eight variants were significantly different between Thais and one or more populations. Three of the eight variants (rs4986893, rs79527462, and rs3892097) were not different between Thais and East Asians but different to the other four populations.

**Table 1:** The novel variants (which were not reported in 1000 Genomes Project Phase 3) with high impacts identified in the 171 individuals.

Novel	Genes	Chr	Positions	Reference Allele	Alternate Allele	Allele frequency
1	*CACNA1S*	1	201016674	G	A	0.0058
2	*CFTR*	7	117230480	G	T	0.0029
3	*CYP1A1*	15	75013810	G	GATGGCGACGTACAATCTTTTCAGAAACATCATTAAGAACATCATCAAATGTAAAATAACCATCCTCTGAAGCGAGTTGA	0.0029
4	*CYP2A13*	19	41600328	C	CCTCCCTAA	0.0029
5	*CYP2B6*	19	41497355	AG	A	0.0029
6*	*CYP2C19*	10	96602646	C	A	0.0029
7	*CYP2D6*	22	42523521	C	CCCAAA	0.0029
8	*CYP2D7*	22	42537234	C	CCCAAA	0.0058
9	*CYP2D7*	22	42538514	T	TC	0.4152
10	*CYP2F1*	19	41627939	G	GA	0.0029
11	*CYP2W1*	7	1024201	C	T	0.0029
12	*CYP2W1*	7	1024653	CCT	C	0.0029
13	*CYP3A5*	7	99250232	A	C	0.0029
14	*CYP4F2*	19	15997044	GCCCTCA	G	0.0205
15	*SLC22A2*	6	160671579	C	T	0.0029
16	*SLCO1B1*	12	21294593	G	A	0.0029
17	*SLCO1B3*	12	21015371	CAT	C	0.0029
18	*SLCO1B3*	12	21036518	TTATC	T	0.0058
19	*SLCO1B3*	12	21054403	T	C	0.0029
20	*UGT1A4*	2	234627639	CG	C	0.0088
21	*UGT2B7*	4	69962949	TG	T	0.0029

*Novel6 was identified in Singaporean and Malaysian (https://www.ncbi.nlm.nih.gov/projects/SNP/snp_retrieve.cgi?subsnp_id=657184749) and was annotated as rs370320936

Another variant (rs71358943) was not different among Asians (Thais, East Asian, and South Asian) but different from African, admixed American, and European. Two variants (rs61469810 and rs3833221) had the same allele frequencies among populations except for African, which had a much higher frequency. For rs3215983 and rs11935951, frequencies varied across populations where Thais were different from African, South Asian, and European at rs3215983 and different from African and admixed American at rs11935951.

## Discussion

This study reported the star allele profile, diplotypes, and the corresponding predicted phenotypes of 51 pharmacogenes using WGS of 171 unrelated healthy Thais. Compared to the previous report of pharmacogenes in a Thai population recruiting participants with Brugada syndrome endemic in Thailand’s Northeastern region [6], our study enrolled participants from all regions of Thailand (S1 Table in S2 File). However, participants were recruited from one hospital, King Chulalongkorn Memorial Hospital, in Bangkok. This mainly results from the fact that all 20 clinical geneticists except one are in Bangkok [16, 17]; therefore, participants enrolled from the Genetics Clinic of the Hospital, a tertiary referral center were from throughout the country. In addition, 26 more genes were added to the 25 genes in the previous study [6]. These newly studied genes are associated with drugs approved by the Thai Food and Drug Administration, Ministry of Public Health, Thailand (https://porta.fda.moph.go.th) and are used as the treatment for the top 10 diseases in Thai people (S4 Table in S2 File).

Of the 51 studied genes, 40 had at least one individual with variant or altered phenotype. This highlights the need for dose alteration of a prescribed drug or an alternative treatment. Pharmacogenetic testing would benefit everyone, as our data showed that no one had a normal phenotype in all 51 genes. In addition, the high impact SNPs allele frequencies were compared to the other five populations. Twenty variants identified have not been reported elsewhere. Therefore, this study provides genes that should be included in a preemptive pharmacogenetic panel for the Thai population.

The thirteen genes where more than 25% of our samples had variant or altered phenotype is involved in the absorption, distribution, metabolism or excretion of various drugs. Therefore, the need for a change in clinical management depends on the genes and drugs. On the contrary, for some genes, e.g., *CYP26A1*, *CACNA1S*, or *TBXAS1*, the majority of the Thai cohort has a normal phenotype.

One hundred sixty-six individuals had either poor- or decreased-function phenotype in *SLCO1B3* due to *S1. The core SNPs of SLCO1B3*S1 is rs7311358 (c.699G>A). One study analyzed these SNPs in combination with rs4149117 (c.334T>G) and found that rs7311358 AA+AG genotypes and the rs4149117 GG+GT genotypes were associated with a higher probability of not responding to the standard dose of imatinib as a treatment in chronic myeloid leukaemia [18]. In addition, carriers of rs4149117(G)-rs7311358(A) haplotype had a significantly lower uptake of mycophenolic acid glucuronide into the cells than the reference haplotype [19].

*CYP3A*, one of the major CYP families, encodes for various catalyzing enzymes involved in many drug metabolism including nifedipine, cyclosporine, tacrolimus, erythromycin, midazolam, alprazolam, and triazolam [20, 21]. For *CYP3A5*, the CYP3A5*3 allele results in the absence of protein expression due to an improper mRNA splicing [22]. CYP3A5*3 (rs776746) was highly present in Caucasians (85–95%), Mexicans (75%), and Asians (65–85%) and to a lower degree in African Americans (27–55%) [23]. The number of Asians agrees with our results (66.7%). Therefore, to achieve therapeutic drug concentrations, an intermediate metabolizer phenotype such as CYP3A5*1/*3 was recommended to receive an increased dose of tacrolimus (1.5–2 times higher than standard dosing). In contrast, poor metabolizer phenotype such as CYP3A5*3/*3 should receive the standard dosing of medication based on the tacrolimus package insert [24].

*CYP2C19* is well known for the metabolism of particular substances such as omeprazole, imipramine, and diazepam. CYP2C19*17 is mainly found in the European, African, and admixed American, while CYP2C19*2 is commonly found in Asia [2]. In our study, CYP2C19*2 frequency was also high. The homozygous poor metabolizer diplotype (including CYP2C19*2/*2) patients showed an efficient eradication of *Heterobacter pylori* when treated with omeprazole and amoxicillin since the drug remains in the bloodstream longer than normal phenotype and they can avoid drug resistance during therapy [25, 26]. For citalopram and escitalopram, intermediate metabolizer phenotype such as CYP2C19*1/*2 was recommended to initiate therapy with recommended starting dose while poor metabolizer phenotype such as CYP2C19*2/*2 should receive a 50% dose reduction or select alternative drug not predominantly metabolized by CYP2C19 [27].

*CYP2A6* is involved in the metabolism of a large number of xenobiotic and is responsible for approximately 3% of the drugs metabolized by CYP enzymes. CYP2A6*4, *7, *10, *11, *19, and *35 are highly distributed over East Asians, while CYP2A6*14, *28, and *34 have higher frequencies in Caucasian populations [28]. In our data, 3–12% of the population carried decreased-function alleles (CYP2A6*4, *7, *9, and *10) where their homozygous and heterozygous diplotypes were predicted as the slow metabolizer. Individuals with CYP2A6*4, *7*, *9, and *10 were associated with a reduction in nicotine metabolism [29–31] and reduced metabolism of tegafur compared to *CYP2A6* wild type when treated with S-1 (an oral anticancer agent) [32, 33]. In addition, healthy postmenopausal women with CYP2A6*4, *7, *9 had a lower clearance of letrozole than CYP2A6*1 [34].

*SULT1A1* activity plays a crucial role in the metabolism, bioactivation, detoxification of procarcinogens, deactivating catecholamines, and the sulfate conjugation of steroid hormones, including estrogens [35]. *SULT1A1* is in a region with high repetitive sequences and segmental duplication [36], resulting in a highly polymorphic copy number variant (0–6 copies) [37, 38]. A study in Caucasians reported that 64% of the population had two copies, and 32% had ≥ 3 copies [37]. The increasing copies of *SULT1A1* were negatively correlated with a conversion of estrone-sulfate to estrone ratio in men [37]. SULT1A1*2 was common in Caucasians and African Americans but not in Chinese (allele frequencies of 0.332, 0.294, and 0.08, respectively) [39]. Our data agree with those studies (the frequency of 0.07 and ~23% with two copies).

For the X-linked gene, *G6PD*, men have one copy number of the gene while women have two. Therefore, men will be *G6PD* deficient if they inherit only one mutant gene, but women usually need to inherit two abnormal genes [40]. G6PD deficiency has been recognized as a common inherited enzymopathy where G6PD Viangchan (*51) and G6PD Mahidol variant (*31) is highly prevalent in Thais [25]. Other G6PD variants such as G6PD Canton (*8), G6PD Kaiping (*28), G6PD Union (*50), and G6PD Gaohe (*153) were found in Chinese, Indian and Southeast Asia populations [41]. Most men carried the normal allele in our cohort except eight individuals carried G6PD*8, *21, *28, or *51. The core SNP of G6PD*8 is rs72554665 (g.153760484C>A). A report showed that after ingesting fava beans, a 26-month-old Chinese-Japanese boy carrying rs72554665(A) allele had severe hemolytic anemia [42]. Moreover, rasburicase is contraindicated for males carrying class I, II or III allele e.g., Canton allele due to the risk of acute hemolytic anemia [43].

*CYP4B1* is an interface between the metabolism of xenobiotics such as 2-aminofluorene and endobiotic, including ligands where P450 acts on them to modify endogenous processes [44]. It is predominantly expressed in human lungs and might contribute to carcinogenesis [45]. About 20% of our samples carried CYP4B1*2. A study in Japanese found that individuals with CYP4B1*1/*2 or *2/*2 genotypes had a 1.75-fold increased risk of bladder cancer [46]. The frequencies of the CYP4B1*2 was 0.328 in Japanese and 0.147 in French Caucasians [47].

*CYP2D6* and its pseudogene, *CYP2D7*, are responsible for metabolizing and eliminating >20% of drugs with variability among different ethnicity [48]. CYP2D6*2 and CYP2D6*4 are common in Europeans, Africans, South Asians, and admixed Americans, while CYP2D6*5 (gene deletion) and CYP2D6*10 are dominantly observed in East Asian [2]. In addition, CYP2D6*41 were remarkably found in South Asian [2]. Here, we found that CYP2D6*36+*10 had the highest frequency followed by CYP2D6*10 in our cohort while CYP2D6*2, *4, *5, and *41 were found in a low proportion. For intermediate metabolizers with CYP2D6*10, e.g., CYP2D6*10/*10 and CYP2D6*10/*41, a guideline recommends initiating atomoxetine at 40 mg/day and increasing to 80 mg/day after two weeks if no clinical response and in the absence of adverse events [49].

The expression of human *CYP2F1* is found restrictively in lung tissues with the role of metabolizing inhaled compounds [50]. The no-function allele, CYP2F1*2, caused by silence mutation was reported at a high frequency, while other variants were less common (0.6–7.2%) in French [50]. The CYP2F1*2 was also common in our cohort.

Individuals with *NAT2* slow acetylator genotypes (homozygotes or compound heterozygotes for NAT2*5, *6, or *7) are associated with an increased risk of anti-tuberculosis drug-induced liver injury [51], risk of cotrimoxazole adverse events in patients with systemic lupus erythematosus [52], and risk of sulfasalazine-induced toxicity [53]. However, almost half of our samples were intermediate acetylator.

*SLCO2B1* is expressed in the luminal membrane of small-intestinal enterocytes and might play a role in the uptake of drugs from the intestinal lumen [54]. Intermediate and poor function phenotype in our samples was due to carrying SLCO2B1*S1, an in-frame deletion variant. The SLCO2B1*S1 was a new star allele reported by Lee et al. (2019) and was found in only East Asian samples [12].

Genetic polymorphism of *UGT1A1* is associated with diseases such as Gilbert syndrome (UGT1A1*6/*6) [55], head and neck cancers [56], colorectal cancer [57], and coronary artery disease [58]. While UGT1A1*60 was observed in Asians (Korean, Chinese, and Japanese), African Americans, and European Americans [59], UGT1A1*6 is a common allele in only Asians [60]. Individuals with 1 or 2 alleles of UGT1A1*6 have a higher risk of irinotecan-induced neutropenia [61, 62]. In addition, the UGT1A1*6 allele is associated with an increased risk of hyperbilirubinemia in Thai HIV-infected patients treated with indinavir [63]. Besides UGT1A1*1, UGT1A1*60 had the highest allele frequency in our cohort, followed by UGT1A1*6.

*SLCO1B1* gene encodes for a membrane-bound sodium-independent organic anion transporter protein (OATP1B1) that plays a role in the hepatic uptake of many endogenous and xenobiotic compounds [64]. Our samples’ poor and decreased function phenotypes were primarily due to carrying SLCO1B1*15 or SLCO1B1*17. A guideline for administrating simvastatin for heterozygous (one decrease function allele), e.g., SLCO1B1*1b/*15, *1b/*17 or homozygous variant (two decreased-function allele), e.g., SLCO1B1*15/*15, *15/*17, *17/*17 stated that a lower dose or alternative statin should be prescribed because it implies intermediate/high myopathy risk for heterozygous or homozygous respectively [65]. In addition, SLCO1B1*15 was found to be associated with an increased relative bioavailability of pravastatin [66], exposure to pitavastatin [67], concentrations of repaglinide [68], risk of drug-induced liver injury due to rifampin [69] and associated with decreased clearance of olmesartan [70], metabolism of rosuvastatin [71], transport of atrasentan [72].

Our data confirm similar star allele distribution in both frequency and predicted phenotype to a previous report in the Thai population [6]. The result of *CACNA1S* was the same. Nineteen genes (*CFTR*, *CYP2B6*, *CYP2C8*, *CYP2C9*, *CYP2C19*, *CYP2D6*, *CYP3A4*, *CYP3A5*, *CYP4F2*, *GSTP1*, *NAT1*, *NAT2*, *NUDT15*, *RYR1*, *SLCO1B1*, *TPMT*, *UGT1A1*, *UGT1A4*, and *VKROC1*) had a very similar pattern for both allele frequency and phenotype. However, three genes (*DPYD*, *G6PD*, and *IFNL3*) showed different findings. DPYD*S12 had the highest allele frequency in our study resulting in intermediate metabolizer phenotype, while DPYD*1 had the highest allele frequency, and DPYD*S12 was not found in the previous study [6], resulting in normal phenotype shown. We detected many star alleles in *G6PD*, i.e., G6PD*1x2, *21, *28, *31, *50, *51, *8, and *DEL resulting in intermediate metabolizer phenotype while the previous study [6] detected only G6PD*1. For *IFNL3*, both studies detected IFNL3*1 and IFNL3*S3. However, we found IFNL3*DEL in one haplotype and the predicted phenotype was different. We reported unfavorable responses while the previous study reported rapid metabolizer phenotype. Since our results for *GSTM1* and *UGT2B15* might not be reliable, we did not compare the results for these two genes.

The three control genes provide consistent results in our case due to their similar read depths. The important thing to note is that the read depth of a control gene and a calling gene should be comparable; otherwise, a duplication/deletion might be reported if a control gene has a lower/higher read depth. In our study, when results disagree between the three control genes, *RYR1* reported a duplication, whereas the other two control genes reported a normal copy. When we inspected read depth, we found that *RYR1* had a lower read depth than others.

The 51 pharmacogenes we studied were all the genes that Stargazer version 1.0.8 can call. This is the highest number of genes able to be called at the present, compared with other tools, e.g., Aldy [73], StellarPGx [74] and Astrolabe [75] which support 35, 13, and 9 genes, respectively. Sixteen genes had PharmGKB level 1A clinical annotation for specific variant-drug pairs (S2 Table in S2 File). Genes with levels 3 or 4 clinical annotations might be nontrivial as some variants were more frequent in our population than other populations; therefore, the variant-drug pairs might be understudied in our context.

Our data is from a short-read sequencing technique whose accuracy for calling structural variants, e.g., copy number variation, might be suboptimal. This might affect the results of some genes, e.g., *CYP2D6*.

Our study recruited healthy parents of children with rare diseases; thus, more prevalent pathogenic variants for rare diseases are expected, as observed in the Thai Reference Exome (T-REx) Database with similar inclusion criteria [76]. T-REx recruited unaffected parents of children with rare diseases and found a more enriched pathogenic variants, compared with gnomAD. However, this study did not recruit any patients with diseases related to pharmacogenes such as G6PD deficiency. We intentionally recruited only parents of patients with 1) *de novo* mutations such as achondroplasia or 2) diseases unrelated to these 51 pharmacogenes such as methylmalonic academia and Duchenne muscular dystrophy. Therefore, the increased prevalence of the pathogenic variants in these healthy parents or even the patients themselves should not affect this study conclusion.

In conclusion, our study provides pharmacogenomics implications for drug prescription and guidance of population-specific genotyping policy to improve drug response or prevent adverse drug reactions for Thais. In addition, the study provides genes with a high proportion of the population expressing variant or altered phenotypes, which should be included in a pre-emptive pharmacogenomics panel.

## Supporting information

S1 File This file contains S1-S5 Figs. (DOCX)

S2 File This file contains S1-S4 Tables. (DOCX)
