# Supplementary material for: Genotypic and phenotypic landscapes of 51 pharmacogenes derived from whole-genome sequencing in a Thai population
Source: PLoS One. 2022 Feb 17;17(2):e0263621. doi: 10.1371/journal.pone.0263621 (PMC8853512; doi:10.1371/journal.pone.0263621)
Supplement: S1 File — (DOCX) [file pone.0263621.s001.docx]

**S1 File**

**Supporting Figures S1-S5**

**
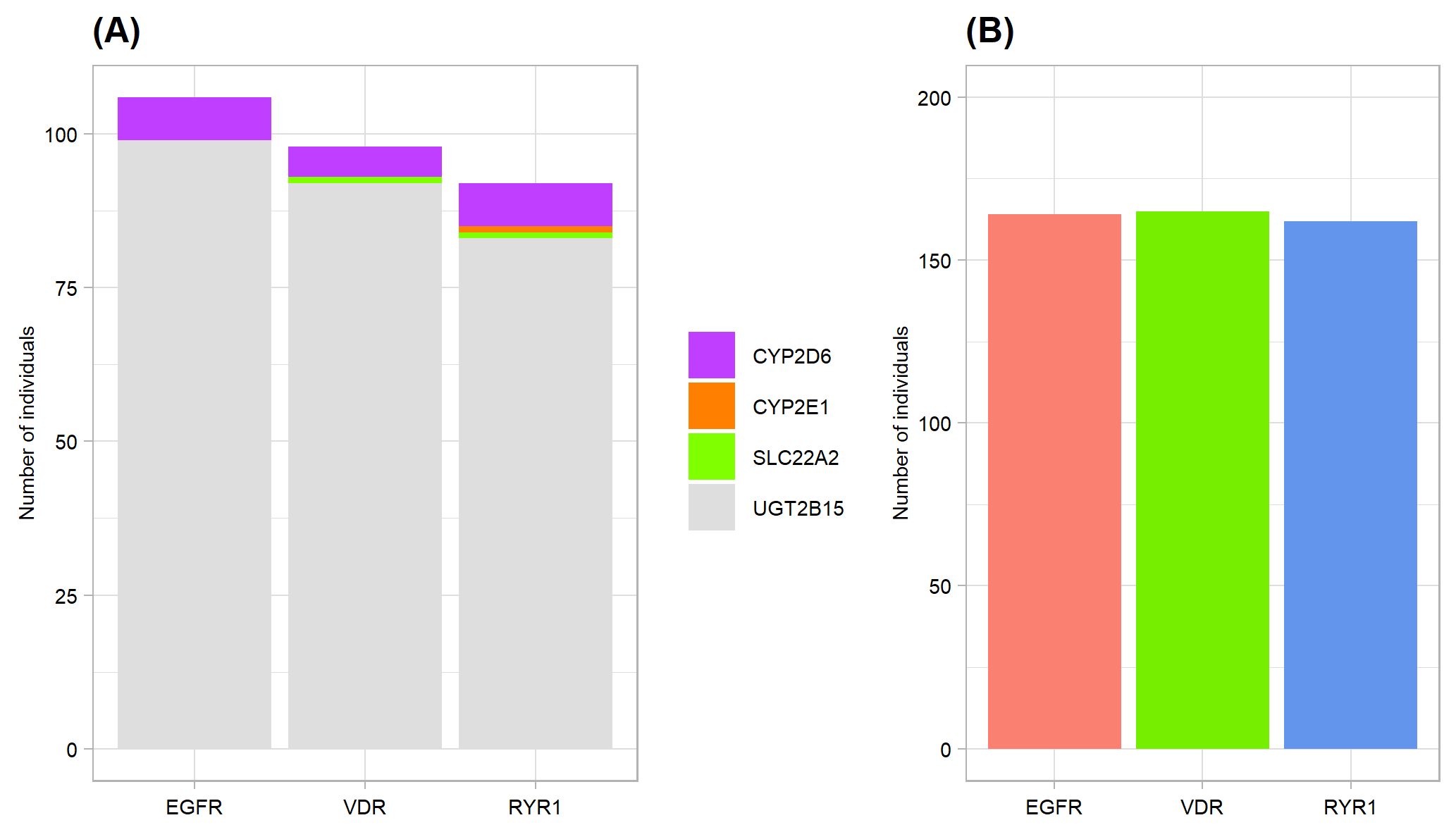
**

**S1 Fig. Comparisons of called results between the three control genes: EGFR, RYR1, and VDR.** (A) The number of individuals with unpredictable genotype in *CYP2D6*, *CYP2E1*, *SLC22A2*, and *UGT2B15* for each control gene. (B) The number of individuals that can be called using each control gene (excluding UGT2B15). Pink, green, blue represents *EGFR*, *VDR*, and *RYR1*, respectively.

(A)


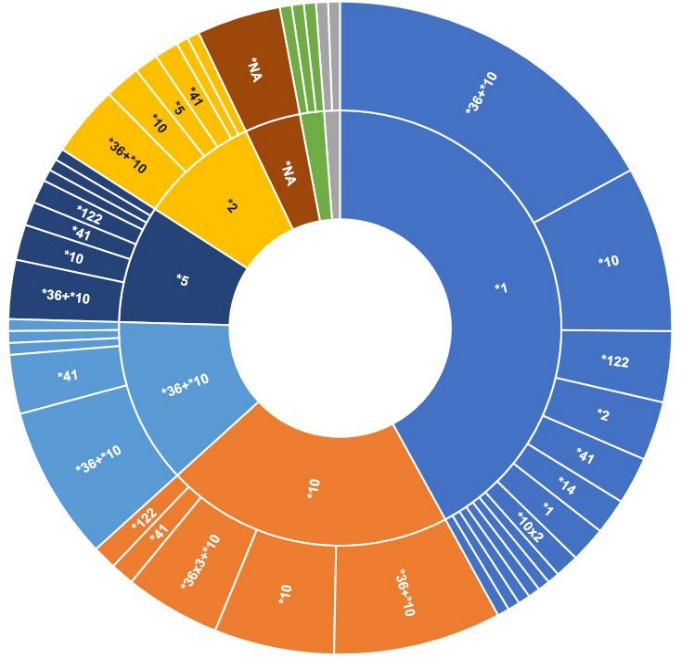


CYP2D6

Phase I

metabolizing genes


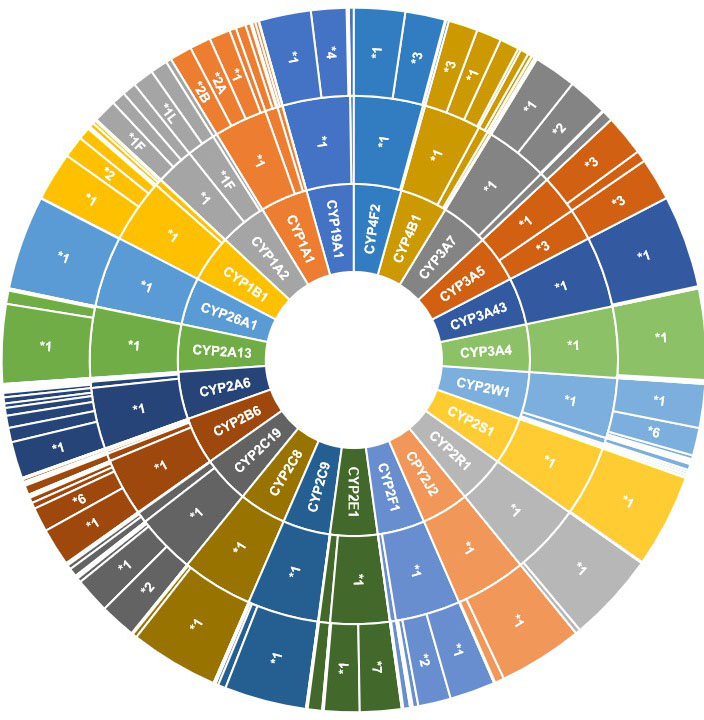


(B)

(C)


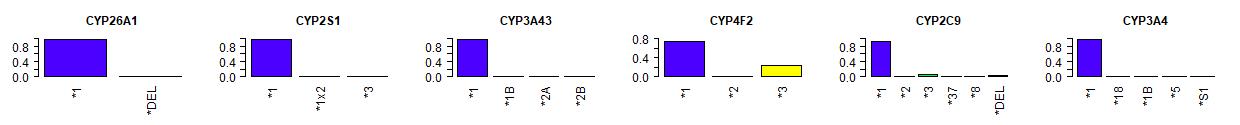

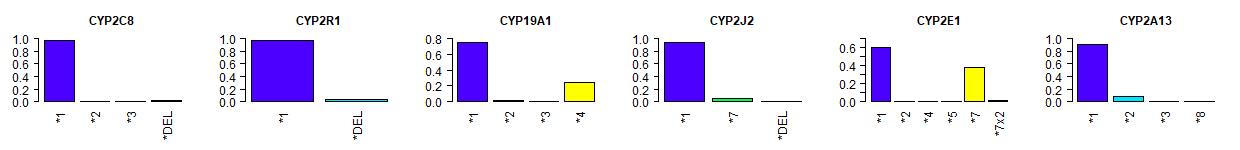

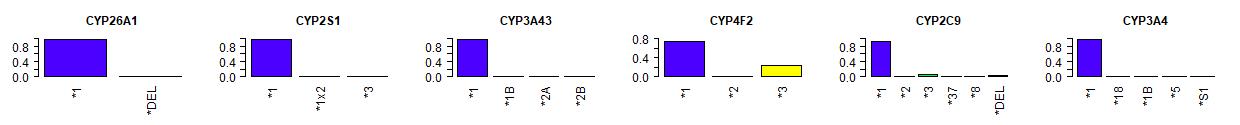

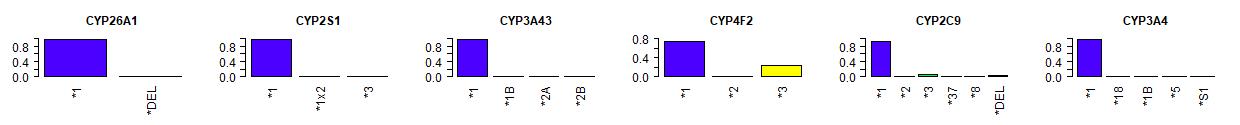

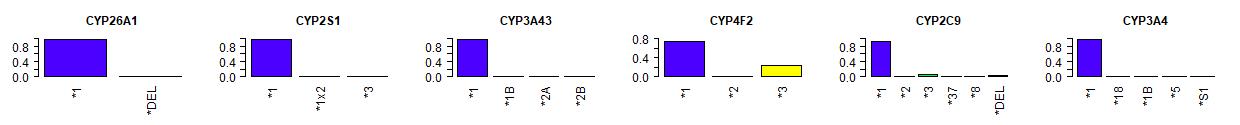

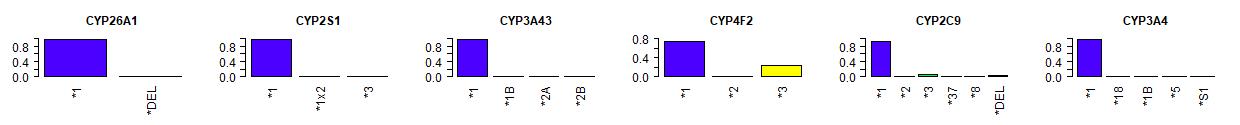

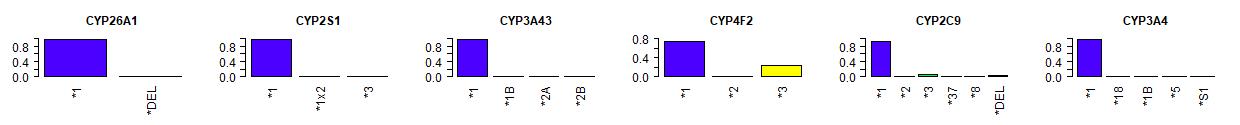

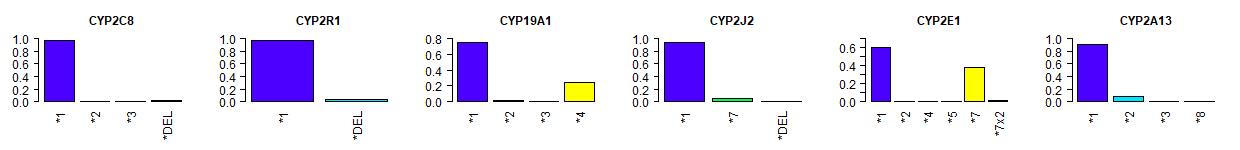

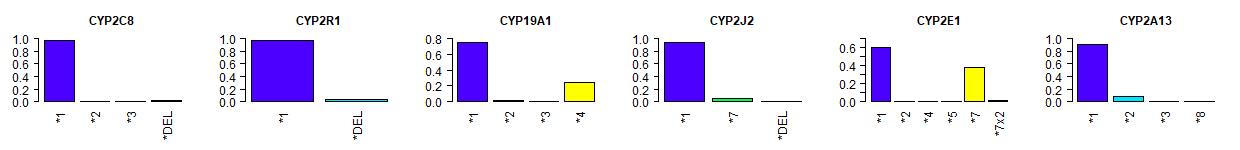

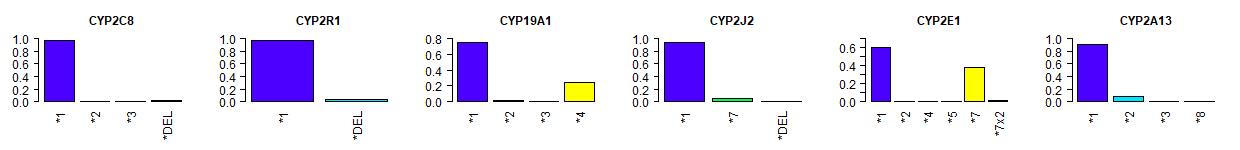

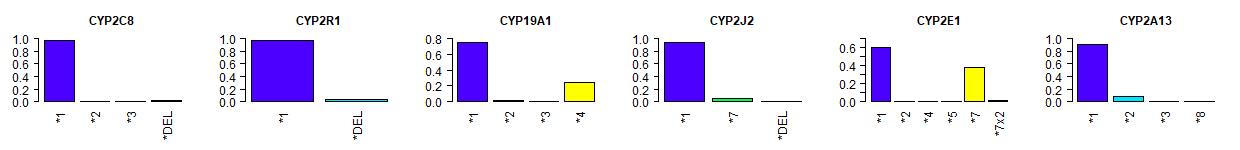

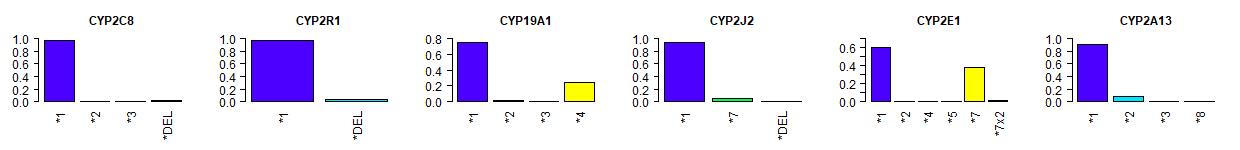

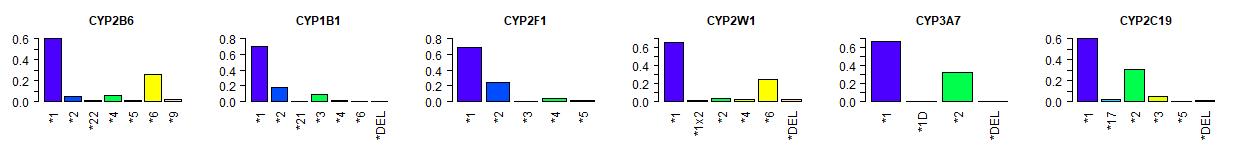

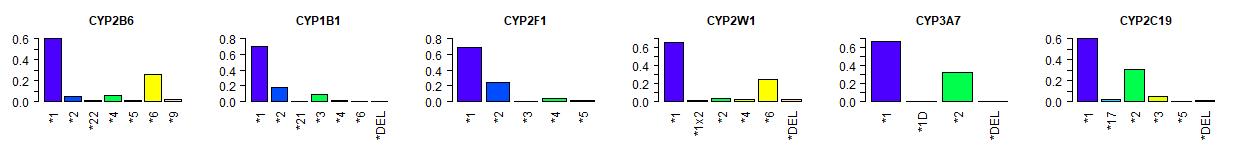

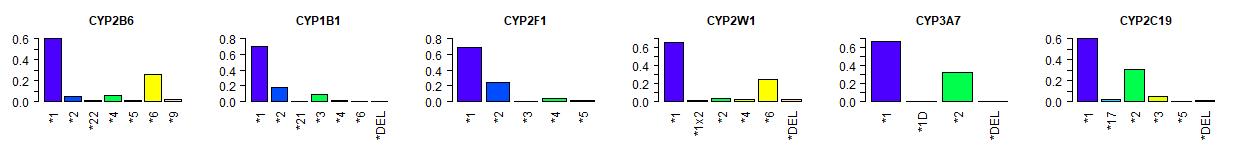

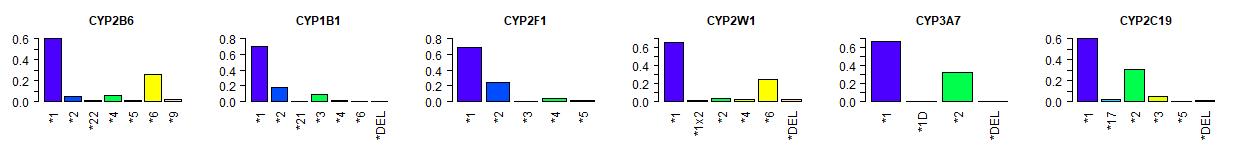

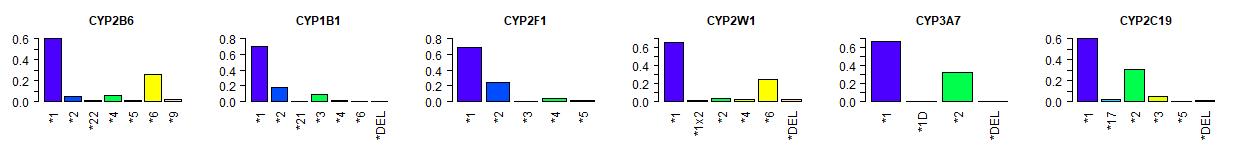

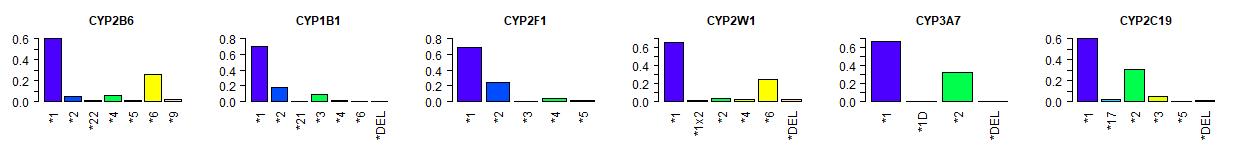

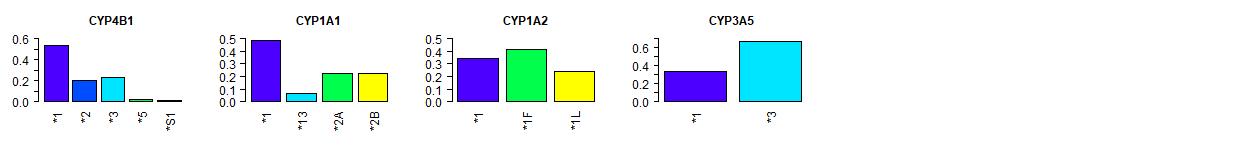

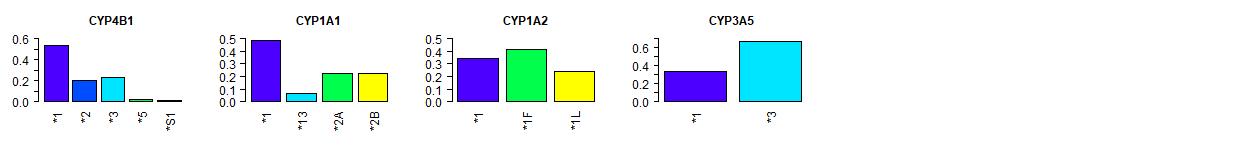

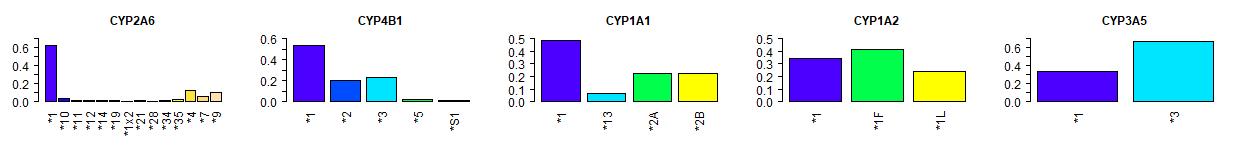

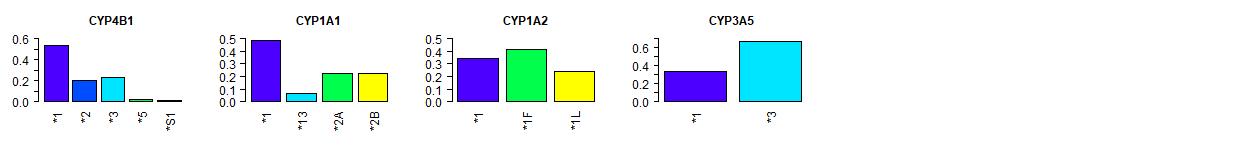

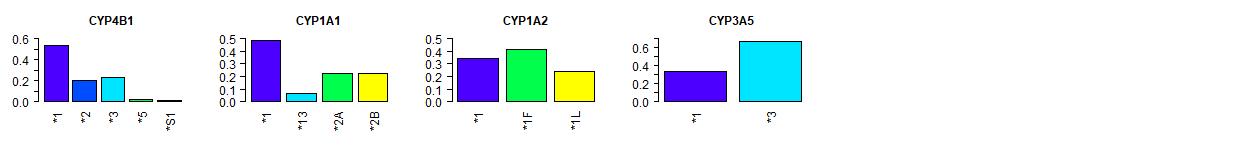


CYP26A1

CYP2S1

CYP4F2

CYP3A43

CYP2C9

CYP3A4

CYP2C8

CYP2R1

CYP19A1

CYP2J2

CYP2E1

CYP2A13

CYP2B6

CYP1B1

CYP2F1

CYP2W1

CYP3A7

CYP2C19

CYP2A6

CYP4B1

CYP1A1

CYP1A2

CYP3A5

Star allele frequency


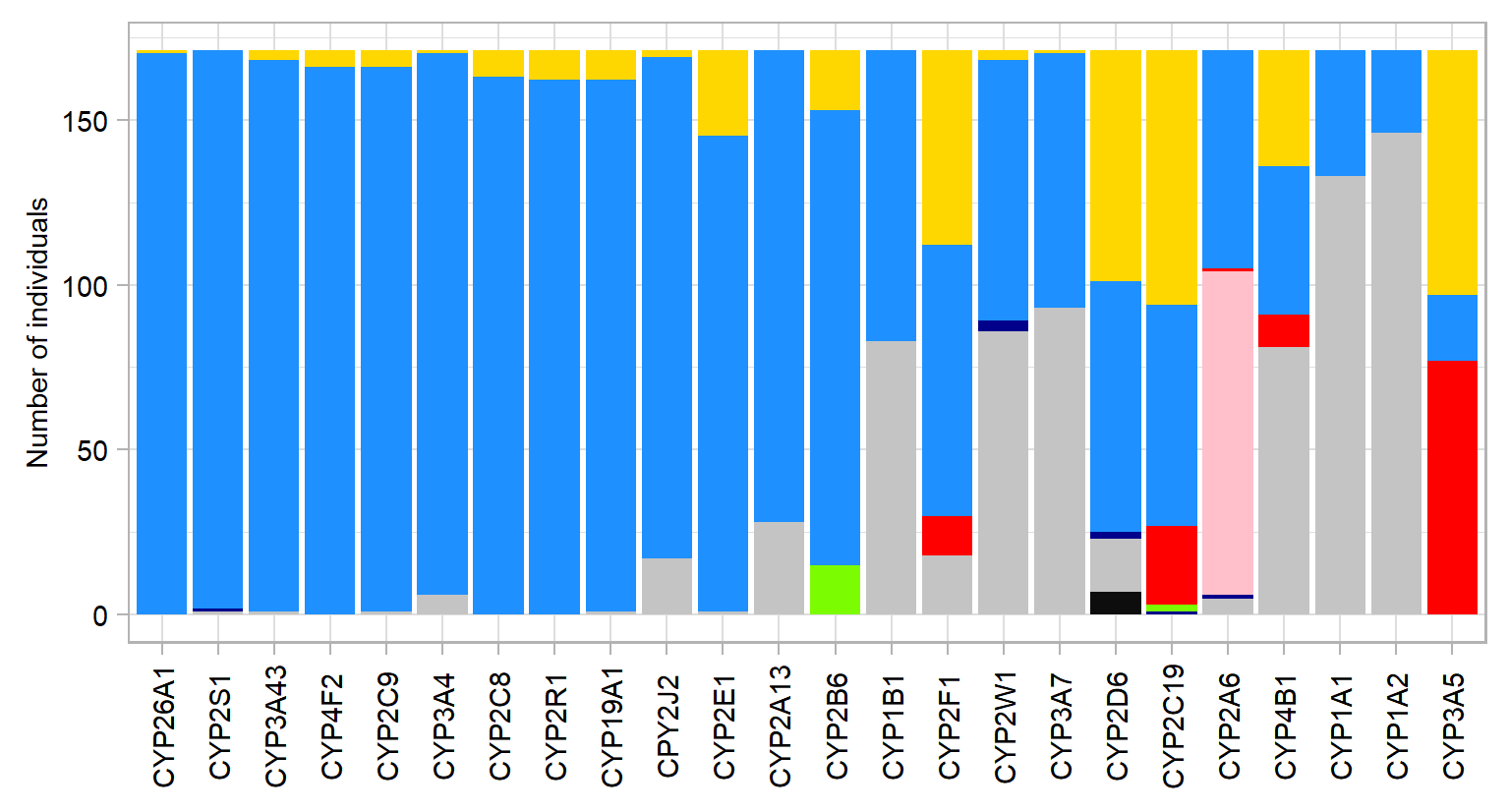


CYP2D6

(D) (E)


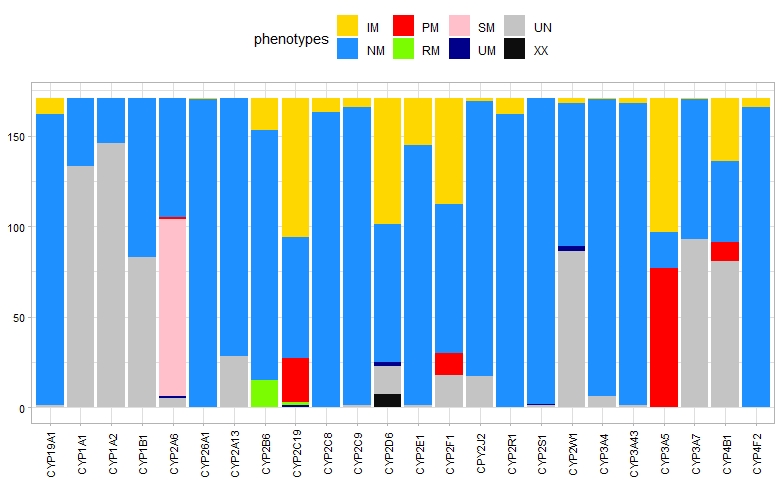


PM


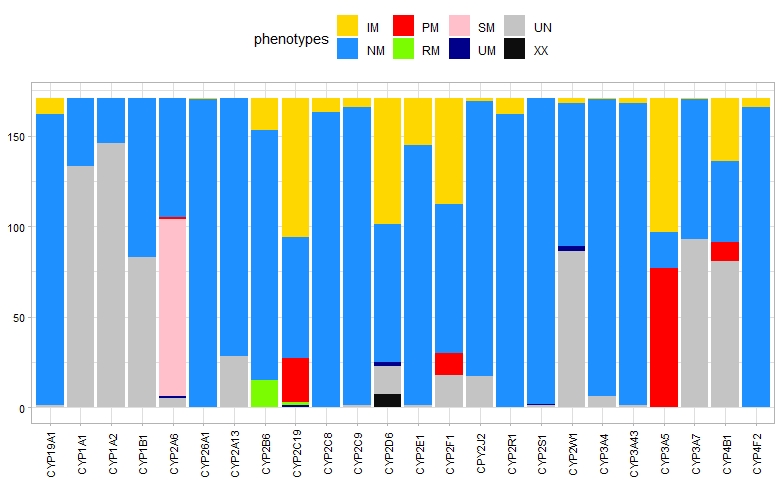


XX


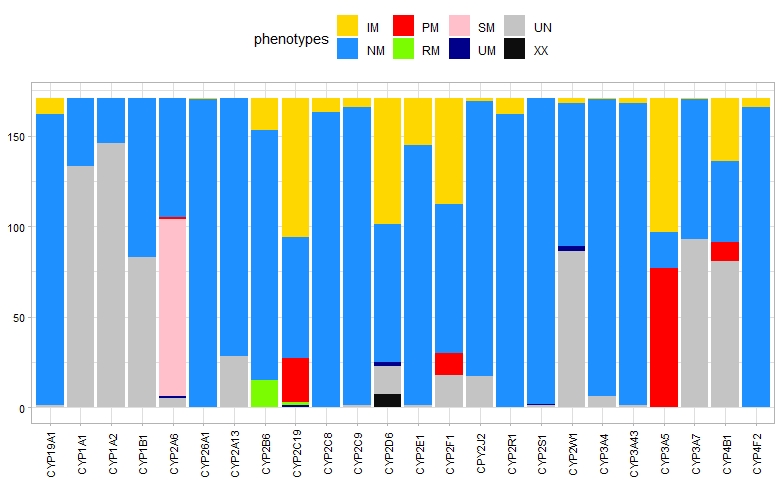


SM


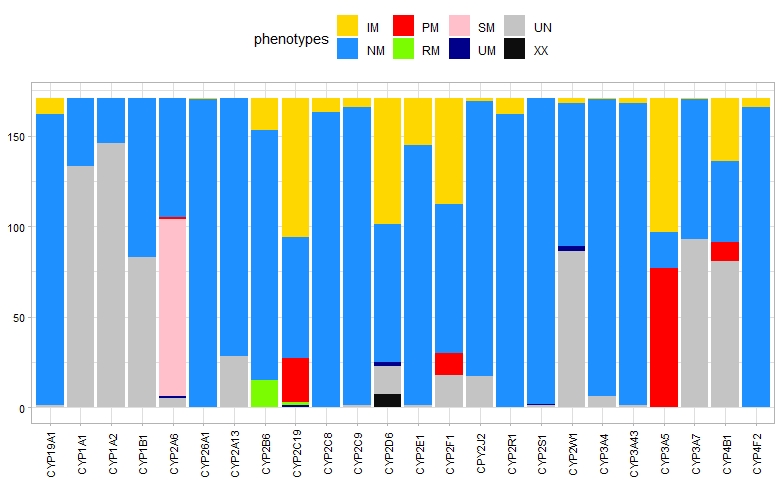


IM


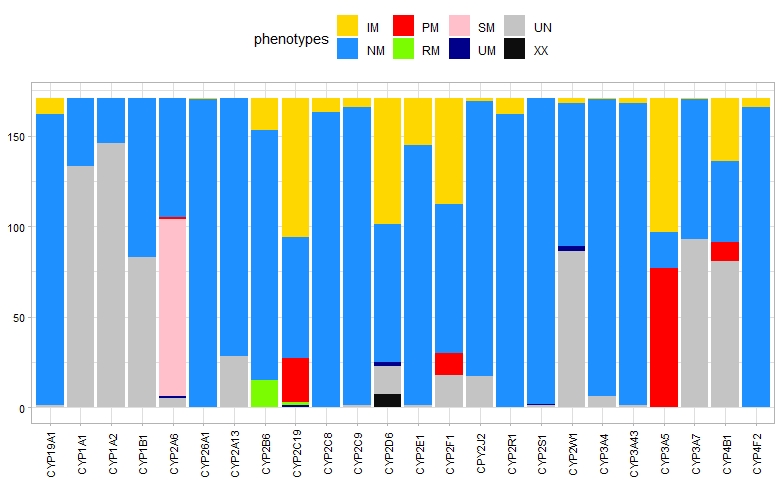


RM


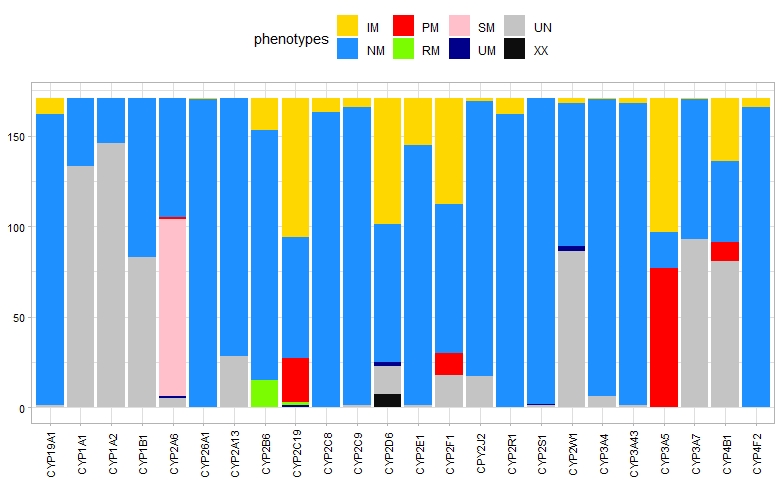


UM


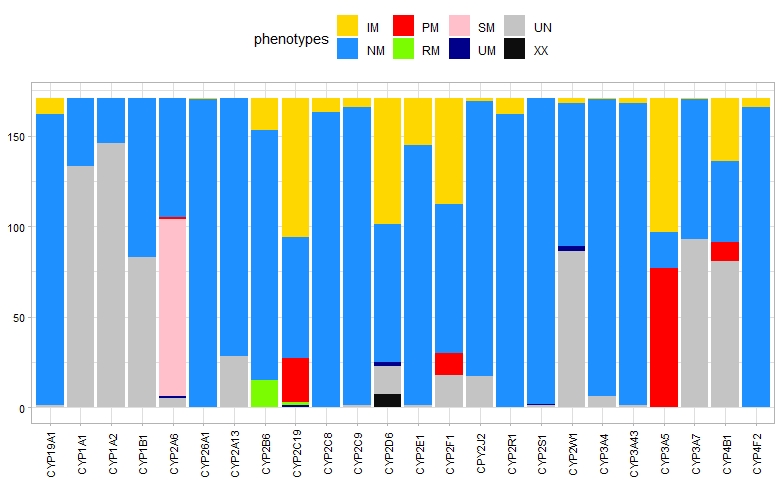


UN


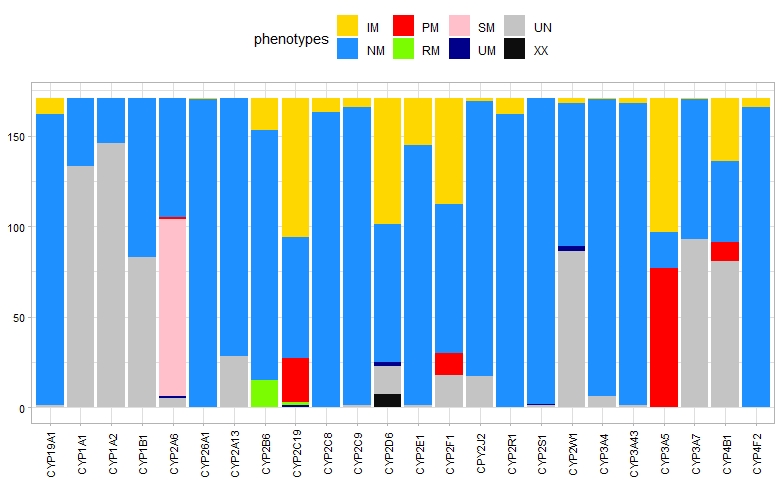


NM


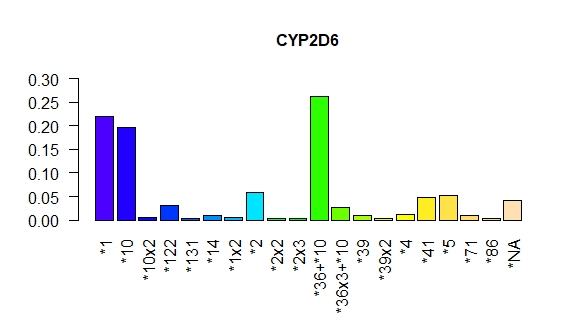


Number of individuals

Star allele frequency

**S2 Fig. Star allele profile and predicted phenotypes of Phase I drug metabolizing enzyme genes.** (A and B) Sunburst plot. The outer ring represents diplotype proportion of each gene, *CYP2D6* and *CYP2A6* (C and D) Bar plot of star allele frequency. (E) The number of individuals for each predicted phenotype. Unpredictable diplotype (XX; black); unknown function (UN; gray); poor metabolizer (PM; red); slow metabolizer (SM; pink); intermediate metabolizer (IM; yellow); normal metabolizer (NM; light blue); rapid metabolizer (RM; green); ultra-rapid metabolizer (UM; dark blue).

(B)


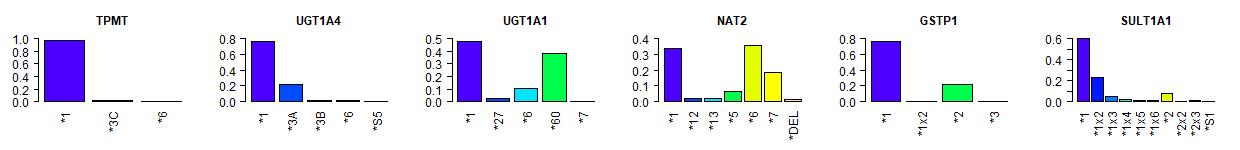

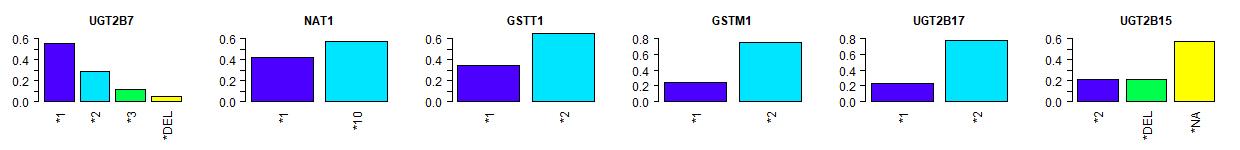

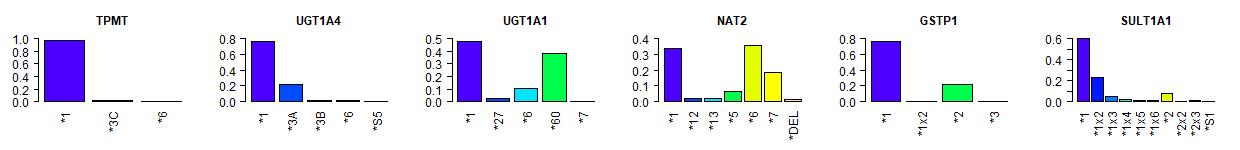

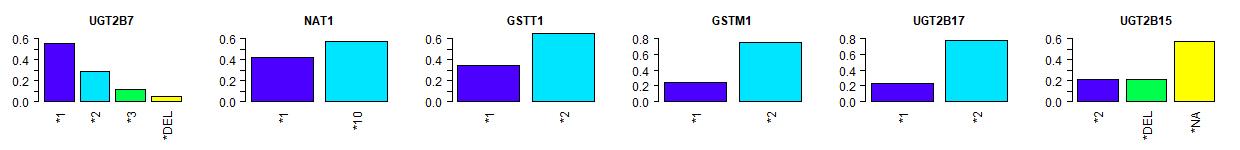


TPMT

UGT1A4

UGT1A1

NAT2

GSTP1

SULT1A1

UGT2B7

NAT1

GSTT1

GSTM1

UGT2B17

UGT2B15

(A)

Star allele frequency


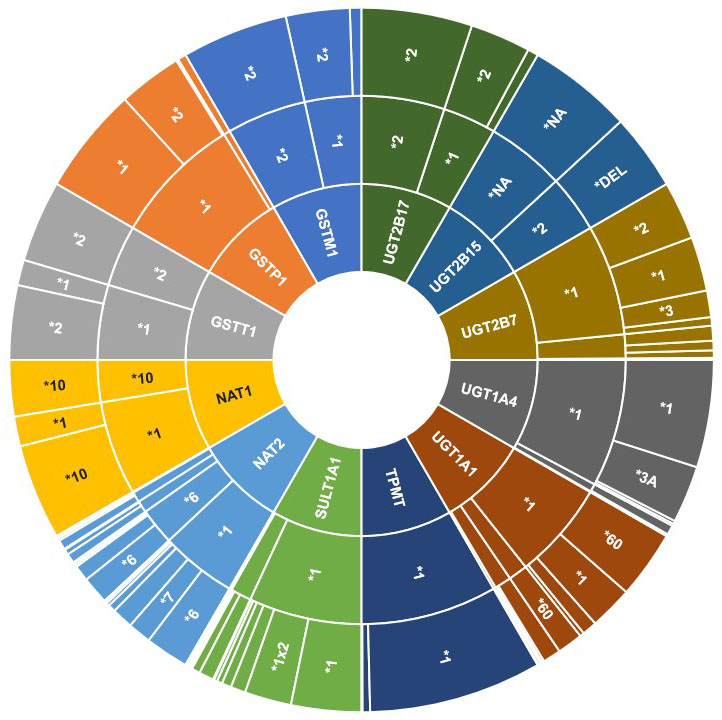


Phase II

metabolizing genes

(C)


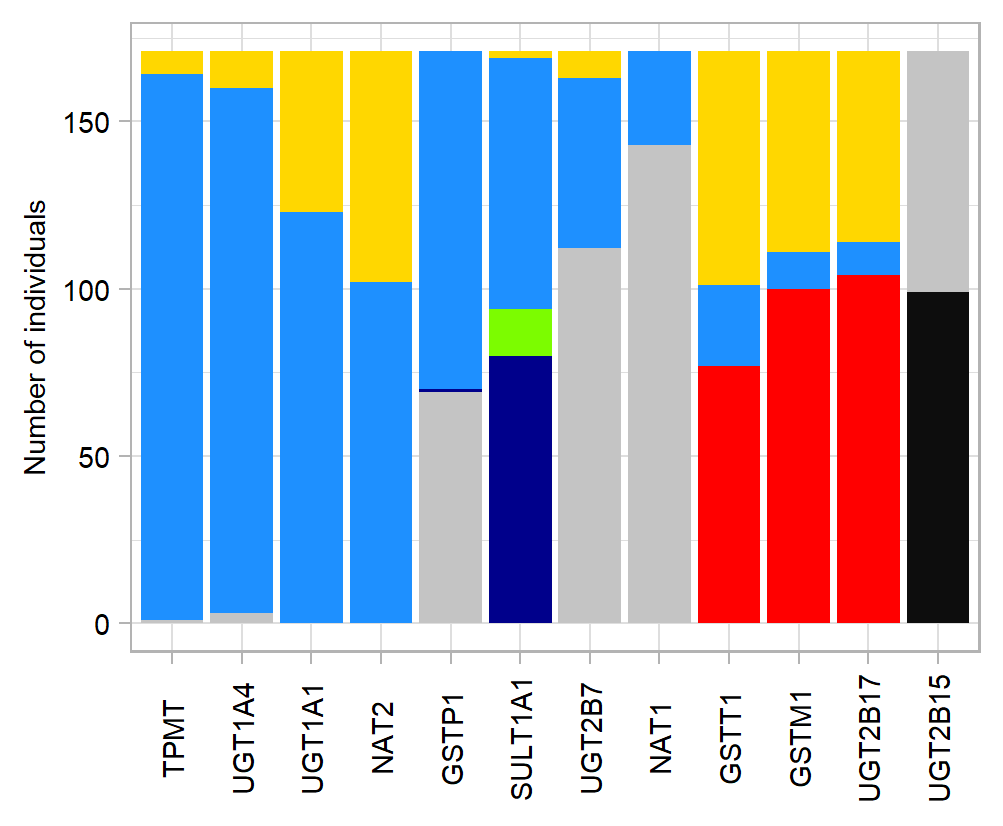

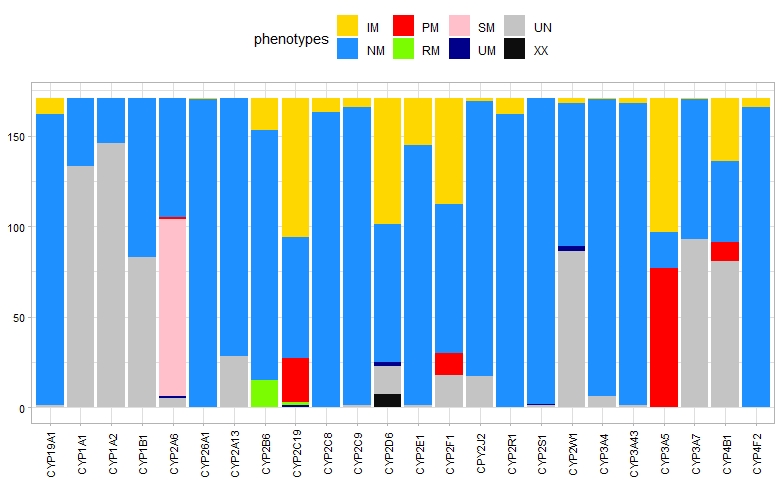


PM


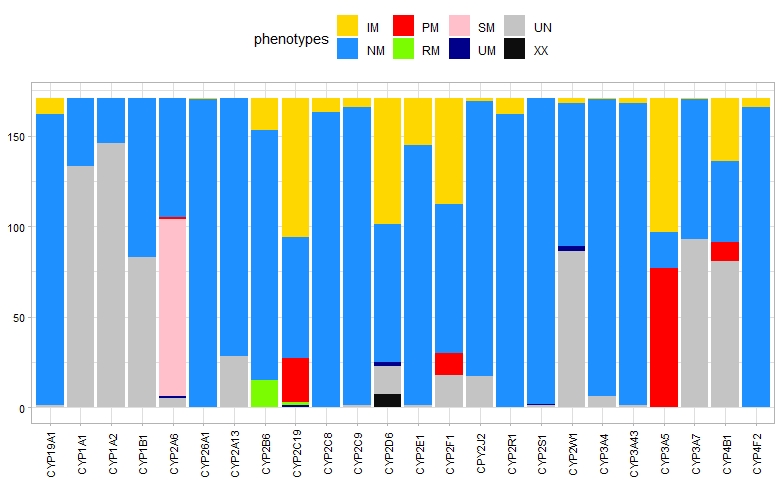


XX


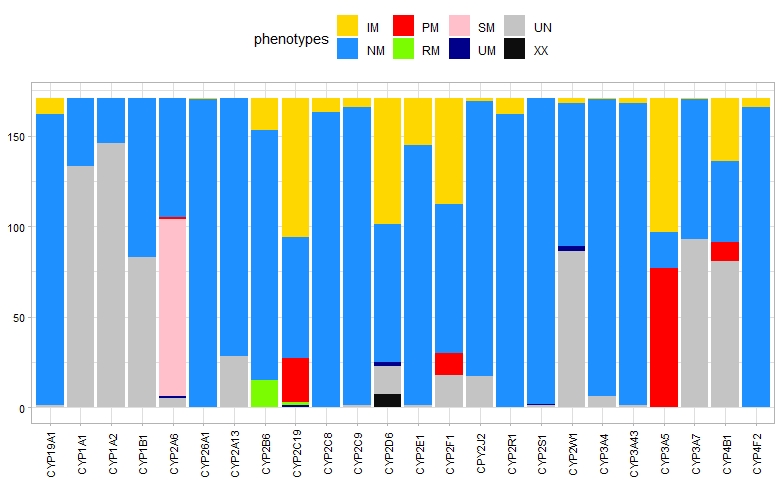


IM


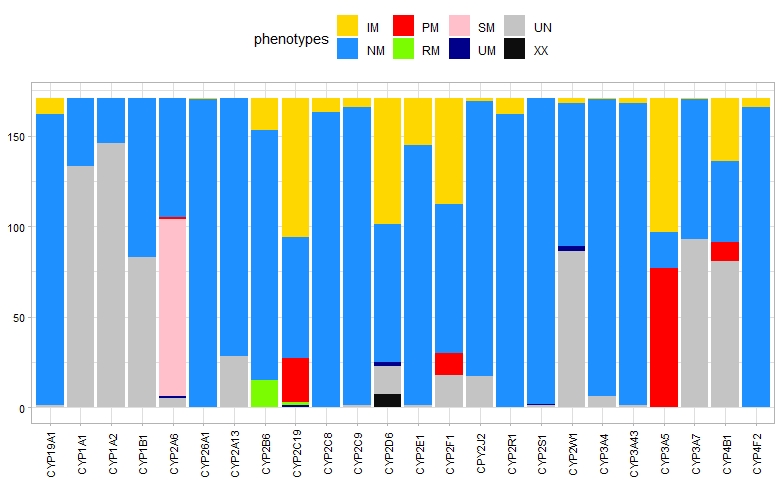


RM


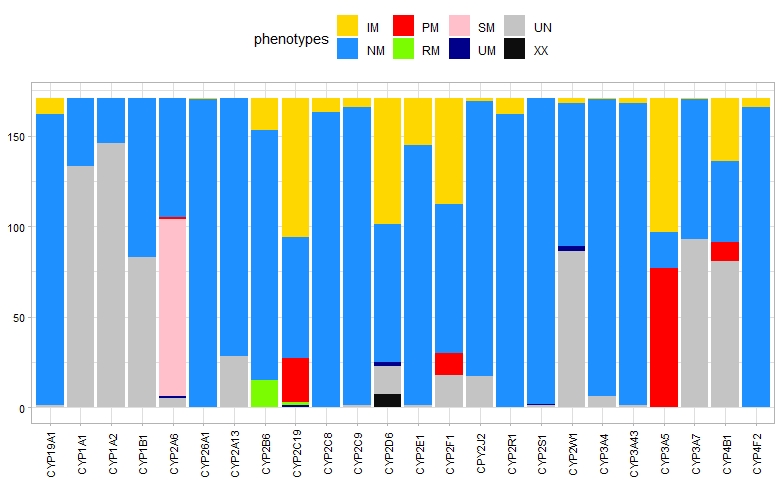


UM


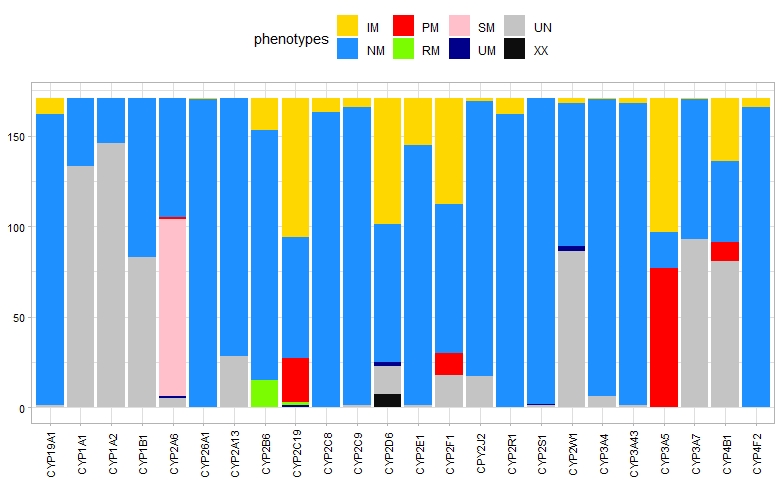


UN


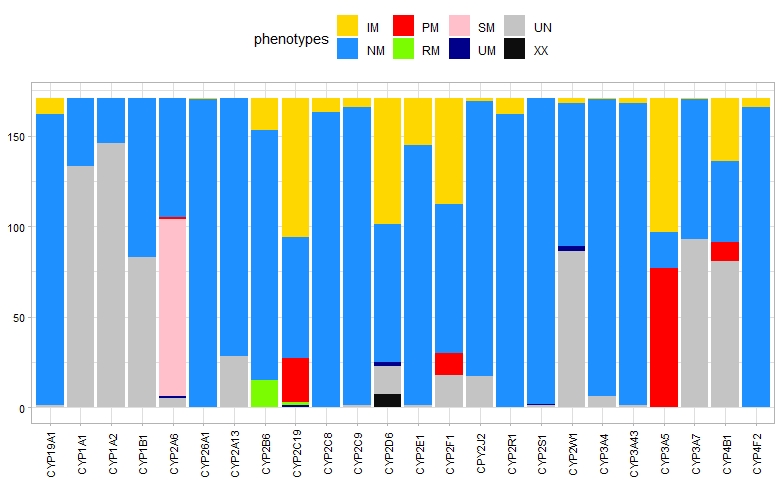


NM

Number of individuals

**S3 Fig. Star allele profile and predicted phenotypes of Phase II drug metabolizing enzyme genes a Sunburst plot.** (A) The outer ring represents diplotype proportion of each gene. (B) Bar plot of star allele frequency. (C) The number of individuals for each predicted phenotype. Unpredictable diplotype (XX; black); unknown function (UN; gray); poor metabolizer (PM; red); intermediate metabolizer (IM; yellow); normal metabolizer (NM; light blue); rapid metabolizer (RM; green); ultrarapid metabolizer (UM; dark blue).


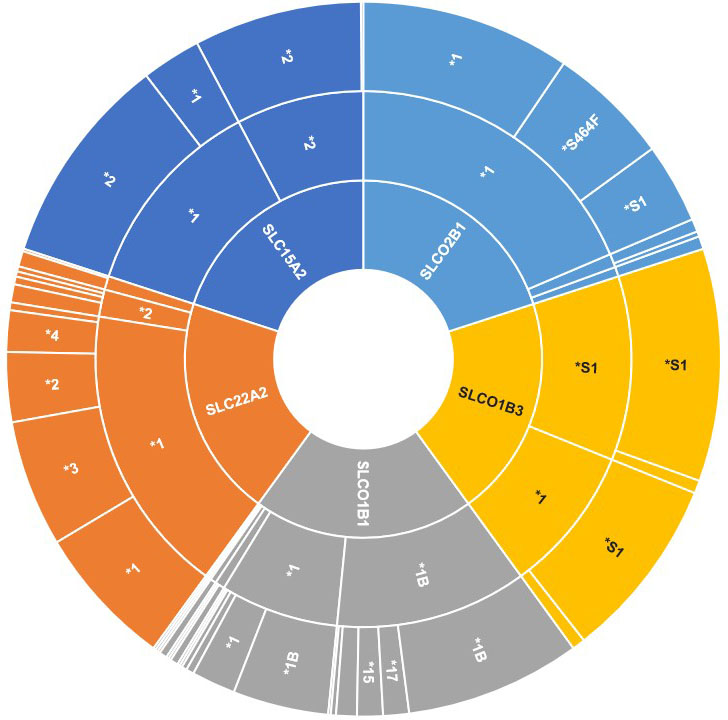
 (B)


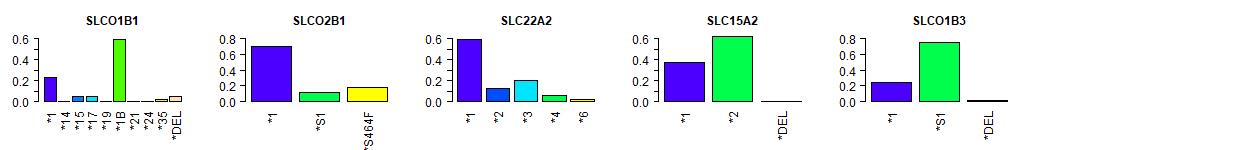

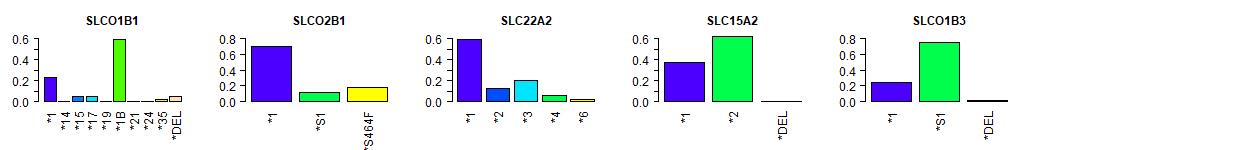


SLCO1B1

SLCO2B1

SLC22A2

SLC15A2

SLCO1B3

(A)

Star allele frequency

(C)


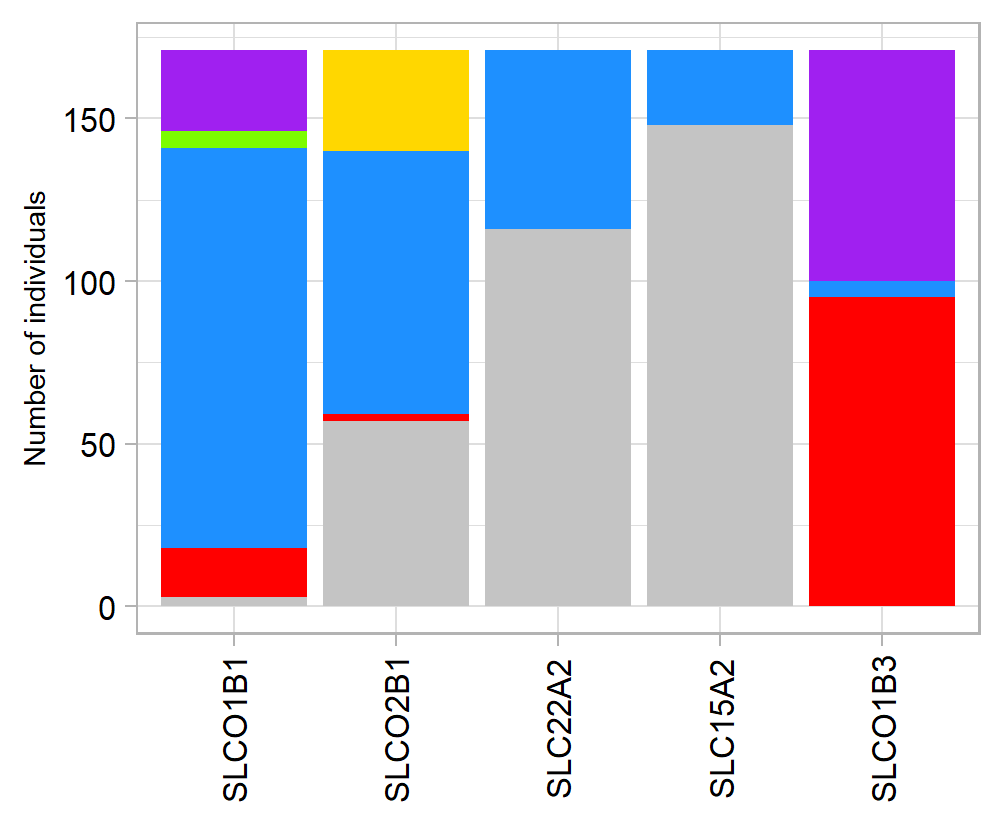


Transporter genes

IF

UN

PF

DeF

NF

InF


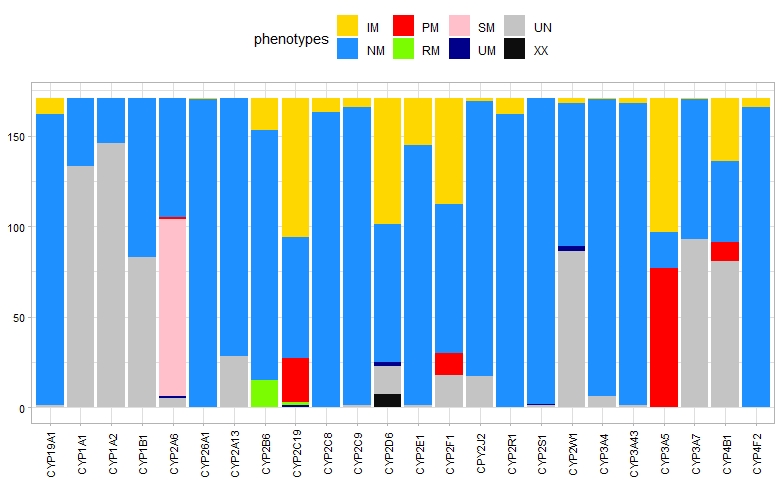

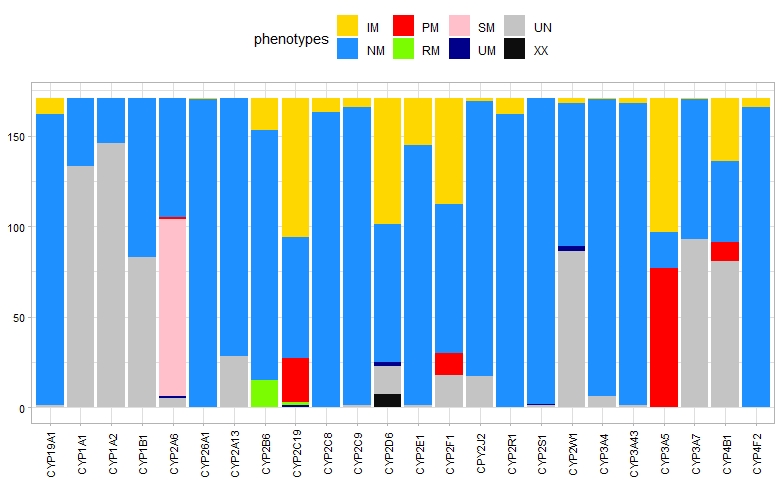

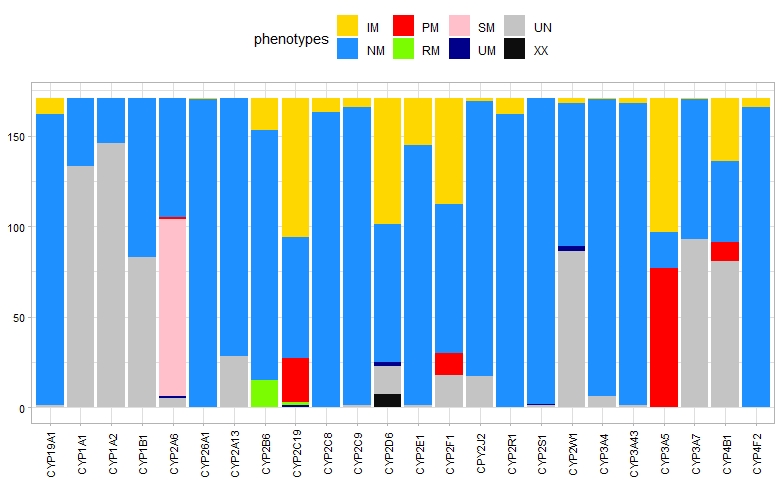

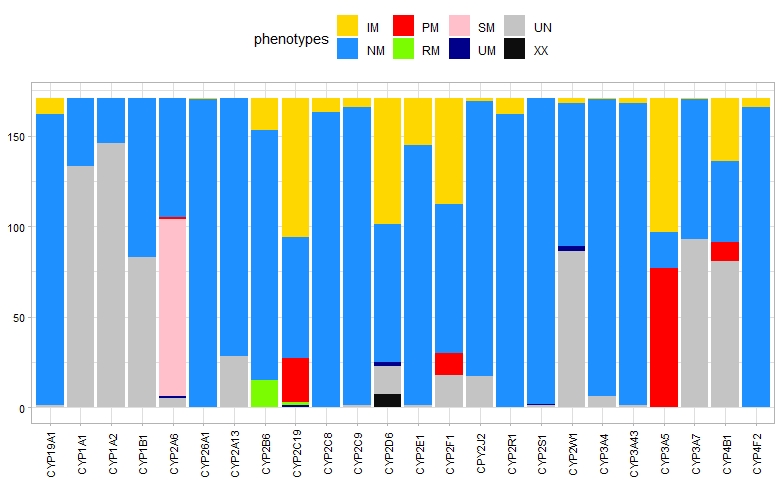

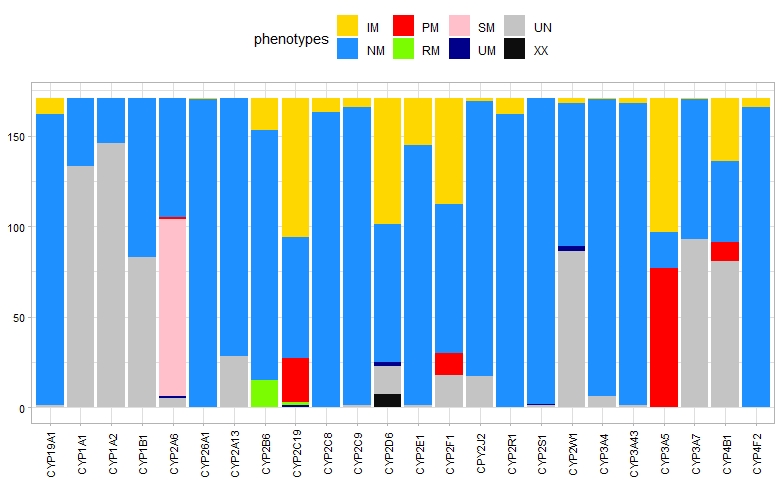

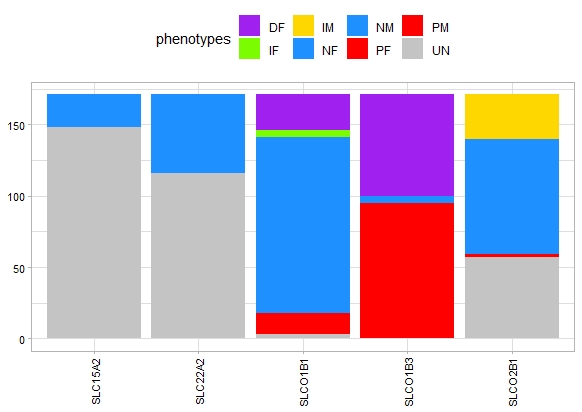


Number of individuals

**S4 Fig. Star allele profile and predicted phenotypes of drug transporter genes.** (A) Sunburst plot. The outer ring represents diplotype proportion of each gene. (B) Bar plot of star allele frequency. (C) The number of individuals for each predicted phenotype. Unknown function (UN; gray); poor function (PF; red); intermediate function (IF; yellow); decreased function (DeF; purple); normal function (NF; light blue); increased function (InF; green).

(B)


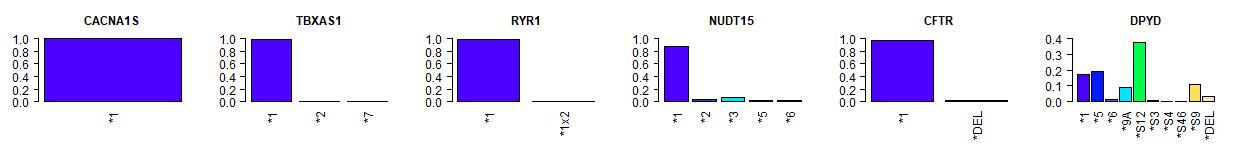

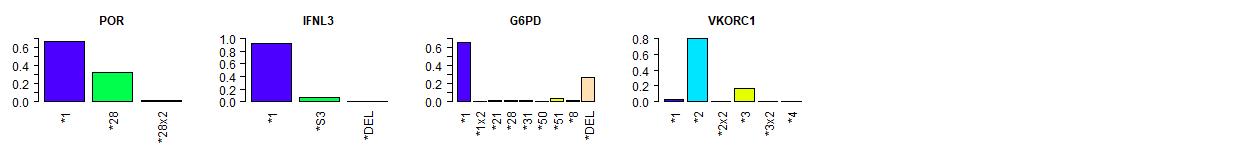

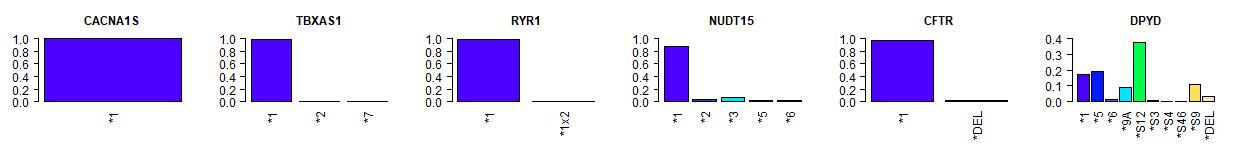

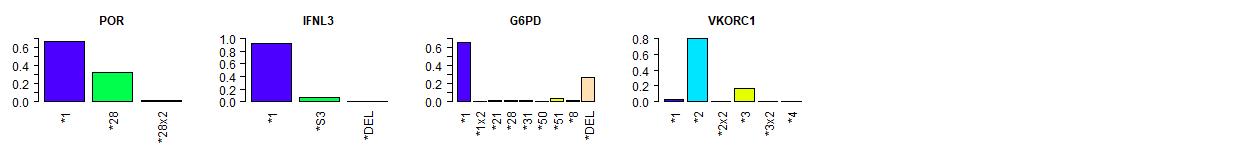


CACNA1S

TBXAS1

RYR1

NUDT15

CFTR

DPYD

POR

IFNL3

G6PD

VKORC1

(A)

Star allele frequency


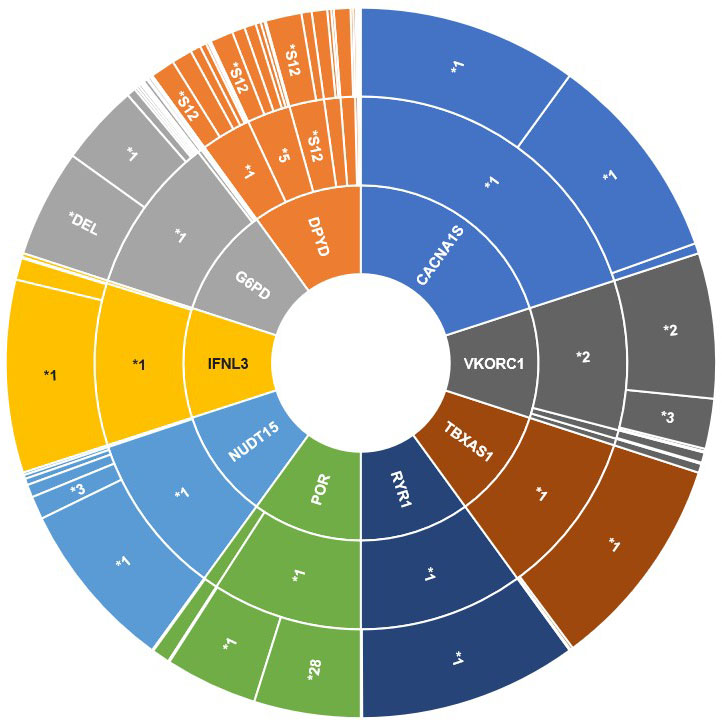


Drug target genes

(C)


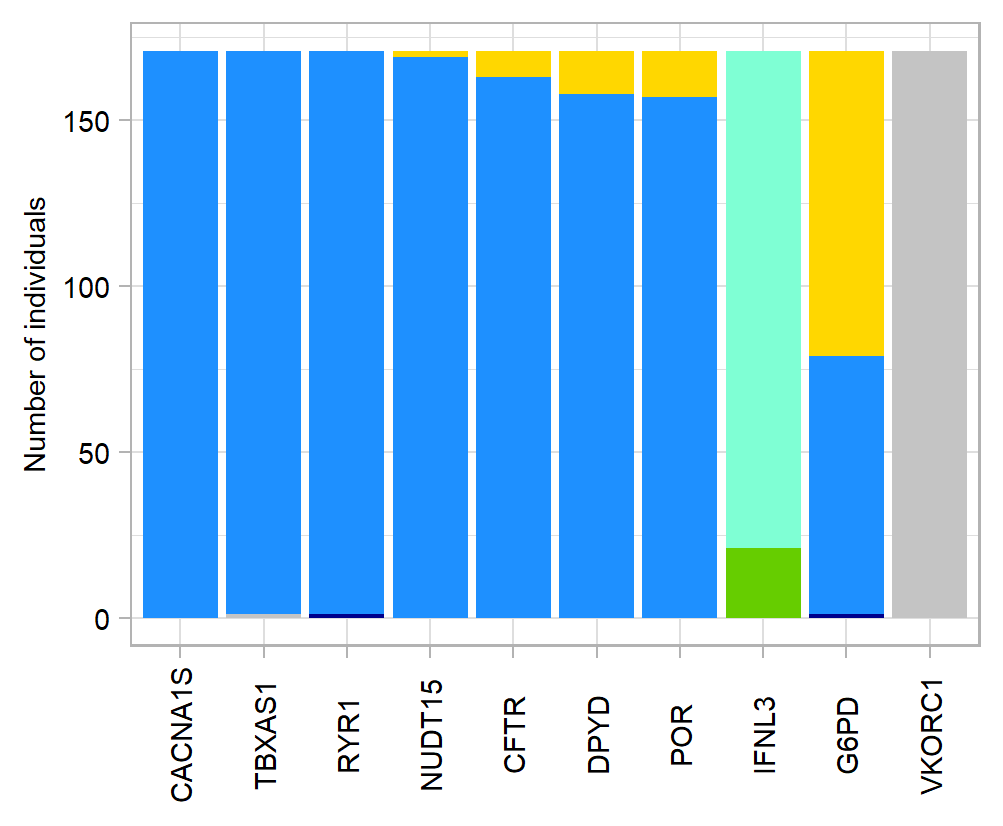


Number of individuals

IM

UN

NM

UM

FR

UR


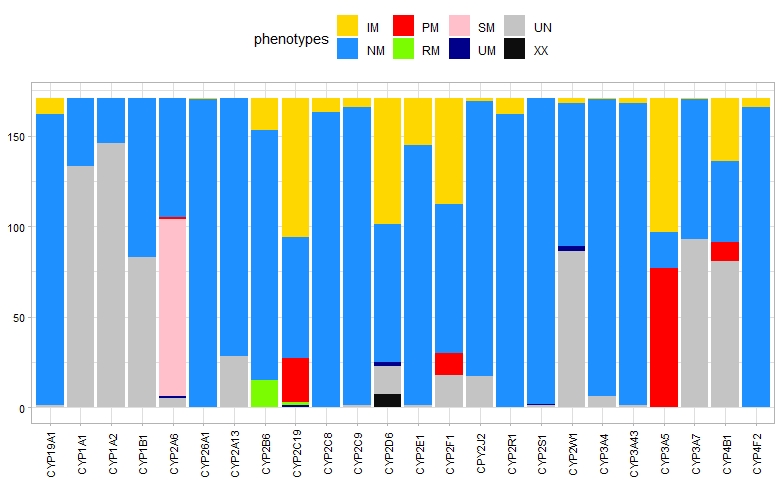

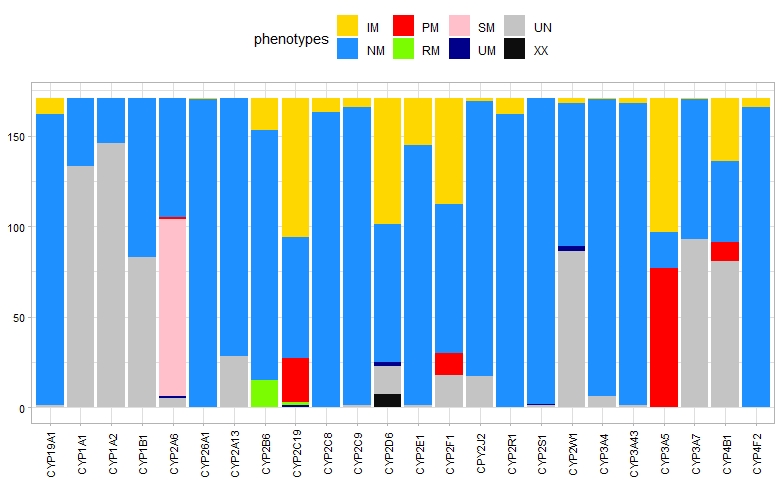

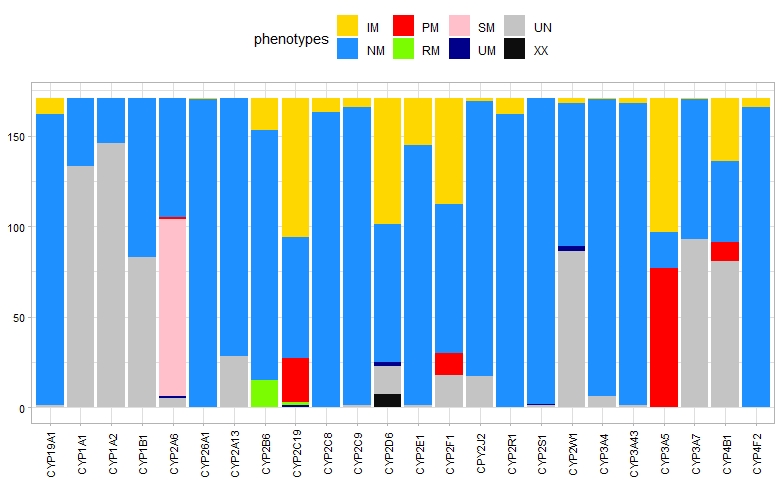

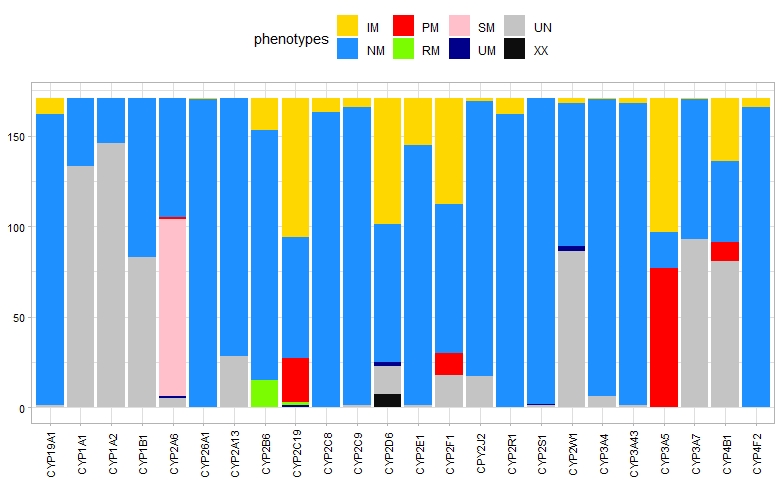

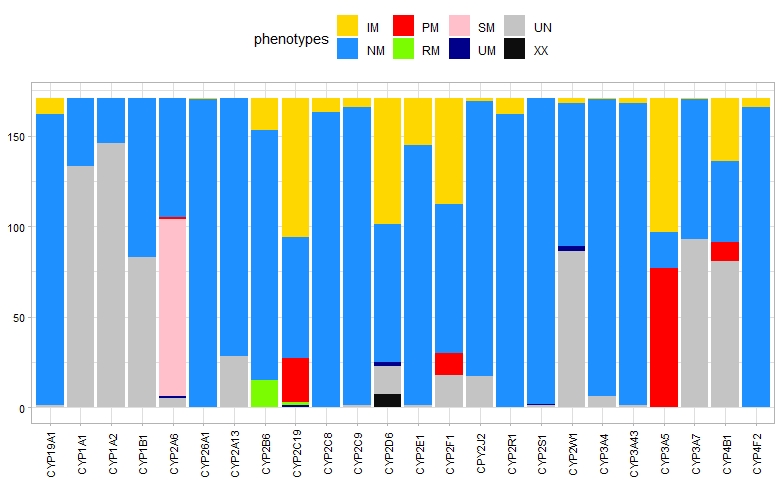

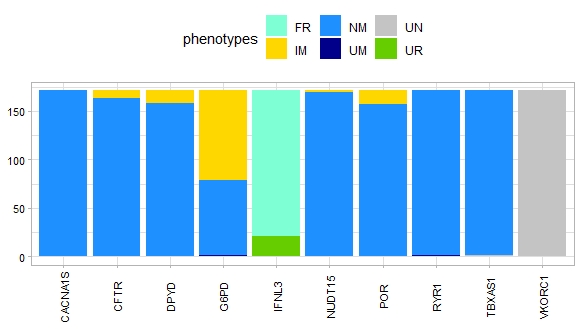


**S5 Fig. Star allele profile and predicted phenotypes of drug target genes.** (A) Sunburst plot. The outer ring represents diplotype proportion of each gene. (B) Bar plot of star allele frequency. (C) The number of individuals for each predicted phenotype. Unknown function (UN; gray); intermediate metabolizer (IM; yellow); normal metabolizer (NM; blue) or favorable response (FR) for *IFNL3* (light blue); unfavorable response (UR) for *IFNL3* (green); ultra-rapid metabolizer (UM; dark blue).
