# Supplementary material for: Genotypic and phenotypic landscapes of 51 pharmacogenes derived from whole-genome sequencing in a Thai population
Source: PLoS One. 2022 Feb 17;17(2):e0263621. doi: 10.1371/journal.pone.0263621 (PMC8853512; doi:10.1371/journal.pone.0263621)
Supplement: S2 File — (DOCX) [file pone.0263621.s002.DOCX]

**S2 File**

**Supporting Tables 1-4**

**S1 Table.** Geographic region of participants.

| **Regions** | **Female** | **Male** | **Total** |
| --- | --- | --- | --- |
| Northern | 3 | 11 | 14 |
| Northeastern | 10 | 5 | 15 |
| Middle | 33 | 31 | 64 |
| Eastern | 5 | 6 | 11 |
| Southern and Western | 3 | 5 | 8 |
| Unknown | 26 | 33 | 59 |
| **Total** | **80** | **91** | **171** |

**S2 Table.** The profile of Star alleles in 51 pharmacogenes with clinical annotation levels of evidence of the 171 healthy Thais.

| **Genes** | **Star alleles (n=196)** | **Clinical annotation levels of evidence** |
| --- | --- | --- |
| **Phase I metabolizing genes (Cytochrome P450) (n=105)** | | |
| *CYP19A1* | *2, *3, *4 | 3 |
| *CYP1A1* | *13, *2A, *2B | 3 |
| *CYP1A2* | *1F, *1L | 3, 4 |
| *CYP1B1* | *2, *21, *3, *4, *6, *DEL | 3 |
| *CYP2A6* | *1x2(dup), *10, *11, *12, *14, *19, *21, *28, *34, *35, *4, *7, *9 | 1B, 3, 4 |
| *CYP26A1* | *DEL | No data |
| *CYP2A13* | *2, *3, *8 | 3 |
| *CYP2B6* | *2, *22, *4, *5, *6, *9 | 1A, 2A, 3, 4 |
| *CYP2C19* | *17, *2, *3, *5, *DEL | 1A, 2A, 3, 4 |
| *CYP2C8* | *2, *3, *DEL | 3, 4 |
| *CYP2C9* | *2, *3, *37, *8, *DEL | 1A, 1B, 2A, 3, 4 |
| *CYP2D6* | *1x2(dup), *10, *10x2(dup), *122, *131, *14, *2, *2x2(dup), *2x3(multi), *36+*10(rea), *36x3+*10(rea), *39, *39x2(dup), *4, *41, *5(del), *71, *86, *NA | 1A, 2A, 3, 4 |
| *CYP2E1* | *2, *4, *5, *7, *7x2(dup) | 3, 4 |
| *CYP2F1* | *2, *3, *4, *5 | 3 |
| *CYP2J2* | *7, *DEL | 3 |
| *CYP2R1* | *DEL | 3 |
| *CYP2S1* | *1x2(dup), *3 | No data |
| *CYP2W1* | *1x2(dup), *2, *4, *6, *DEL | No data |
| *CYP3A4* | *18, *1B, *5, *S1 | 1B, 2A, 3, 4 |
| *CYP3A43* | *1B, *2A, *2B | 3 |
| *CYP3A5* | *3 | 1A, 1B, 2A, 3, 4 |
| *CYP3A7* | *1D, *2, *DEL | 3 |
| *CYP4B1* | *2, *3, *5, *S1 | 3 |
| *CYP4F2* | *2, *3 | 1A, 2A, 3, 4 |
| **Phase II metabolizing genes (n= 38)** | | |
| *GSTM1* | *2(del) | 3, 4 |
| *GSTP1* | *1x2(dup), *2, *3 | 3, 4 |
| *GSTT1* | *2(del) | 3, 4 |
| *NAT1* | *10 | 3 |
| *NAT2* | *12, *13, *5, *6, *7, *DEL | 1B, 2A, 3 |
| *SULT1A1* | *1x2(dup), *1x3(multi), *1x4(multi), *1x5(multi), *1x6(multi), *2, *2x2(multi), *2x3(multi), *S1(del) | 3 |
| *TPMT* | *3C, *6 | 1A, 3, 4 |
| *UGT1A1* | *27, *6, *60, *7 | 1A, 1B, 2A, 3, 4 |
| *UGT1A4* | *3A, *3B, *6, *S5 | 3 |
| *UGT2B15* | *2, *DEL, *NA | 3 |
| *UGT2B17* | *2(del) | No data |
| *UGT2B7* | *2, *3, *DEL | 3, 4 |
| **Transporter genes (n=19)** | | |
| *SLC15A2* | *2, *DEL | 3 |
| *SLC22A2* | *2, *3, *4, *6 | 3, 4 |
| *SLCO1B1* | *14, *15, *17, *19, *1B, *21, *24, *35, *DEL | 1A, 2A, 3, 4 |
| *SLCO1B3* | *S1, *DEL | 3 |
| *SLCO2B1* | *S1, *S464F | 3 |
| **Drug target genes (n=34)** | | |
| *CACNA1S* | None | 1A |
| *CFTR* | *DEL | 1A, 3, 4 |
| *DPYD* | *5, *6, *9A, *S12, *S3, *S4, *S46, *S9, *DEL | 1A, 3, 4 |
| *G6PD* | *1x2(dup), *21, *28, *31, *50, *51, *8, *DEL | 1A, 3, 4 |
| *IFNL3* | *S3, *DEL | 1A, 1B, 2A, 3 |
| *NUDT15* | *2, *3, *5, *6 | 1A, 3 |
| *POR* | *28, *28x2(dup) | 3, 4 |
| *RYR1* | *1x2(dup) | 1A, 3 |
| *TBXAS1* | *2, *7 | 3 |
| *VKORC1* | *2, *2x2(dup), *3, *3x2(dup), *4 | 1A, 1B, 2A, 3, 4 |

*NA = an unpredictable diplotype, dup = gene duplication, del = gene deletion, multi = gene multiplication, rea = gene rearrangement

**S3 Table.** The number of variants corresponding to their impacts and consequences.

| **Impact** | **Consequence** | **Number of known variants** | **Number of novel variants^#^** | **Total** | **Percent** |
| --- | --- | --- | --- | --- | --- |
| HIGH | frameshift_variant | 3 | 12 | 15 | 0.05 |
|  | splice_acceptor_variant | 2 | 0 | 2 | 0.01 |
|  | splice_donor_variant | 4 | 4 | 8 | 0.03 |
|  | stop_gained | 5 | 5^&^ | 10 | 0.04 |
| MODERATE | inframe_deletion | 1 | 6 | 7 | 0.02 |
|  | inframe_insertion | 1 | 3 | 4 | 0.01 |
|  | missense_variant | 233 | 182 | 415 | 1.48 |
| LOW | intron_variant | 34 | 31 | 65 | 0.23 |
|  | stop_retained_variant | 0 | 1 | 1 | 0.00 |
|  | synonymous_variant | 195 | 78 | 273 | 0.97 |
| MODIFIER | 3_prime_UTR_variant | 155 | 147 | 302 | 1.08 |
|  | 5_prime_UTR_variant | 46 | 31 | 77 | 0.27 |
|  | downstream_gene_variant | 1,082 | 973 | 2,055 | 7.32 |
|  | intron_variant | 12,067 | 10,634 | 22,701 | 80.90 |
|  | non_coding_transcript_exon_variant | 3 | 3 | 6 | 0.02 |
|  | upstream_gene_variant | 1,125 | 995 | 2,120 | 7.55 |

^#^novel = not reported in 1000 Genomes Project Phase 3

^&^Of these 5, one, which is Novel6, was identified in Singaporean and Malaysian (https://www.ncbi.nlm.nih.gov/projects/SNP/snp_retrieve.cgi?subsnp_id=657184749) and was annotated as rs370320936

**S4 Table.** A list of diseases and drugs associated with the additional 26 genes.

| **Disease** | **Genes** | **Drugs** |
| --- | --- | --- |
| **Cancer and immunology** | | |
| Neoplasms | *CYP1A1, CYP1A2, CYP2A6, CYP2E1, CYP19A1, CYP1B1, CYP26A1, CYP2F1, CYP2S1, CYP2W1*, *CYP4B1, SLCO1B3, SLC22A2*, *GSTM1*, *GSTP1*, *GSTT1* | capecitabine, erlotinib, docetaxel, tamoxifen, cyclophosphamide, letrozole, antrozole, tretinoin, exemestane, fluorouracil, epirubicine, doxorubicin, sunitinib, cisplatin, paclitaxel, imatinib |
| Immunosuppressant | *SLCO1B3, CYP2J2, CYP3A7,* *GSTM1* | mycophenolic acid, tacrolimus, leflunomide |
| **Non-communicable diseases** | | |
| Cardiovascular diseases | *SLCO2B1, SLCO1B1, SLC15A2, POR, CYP3A7* | ticagrelor, warfarin, simvastatin, atorvastatin, rosuvastatin |
| Other non-communicable diseases | *SLCO2B1, SLC22A2, POR* | metformin, montelukast |
| **Neurological disorders** | | |
| Psychiatric Disorder | *CYP1A1, CYP1A2, CYP3A7, CYP2A6, CYP3A43, UGT2B7, GSTM1, GSTT1* | olanzapine, paroxetine, bupropion, antipsychotics, clozapine, risperidone, ziprasidone |
| Seizures | *CYP1A1, UGT2B7* | carbamazepine, lamotrigine, valproic acid, diazepam |
| **Musculoskeletal disorders** | | |
| Rheumatoid arthritis | *SULT1A1, UGT2B7* | sulfonamides, montelukast |
| **Management of pain and opioid addiction** | | |
| Management of pain | *CYP2A13, CYP2A6, RYR1, UGT2B15, UGT2B17, UGT2B7, TBXAS1* | aspirin, nicotin, oxycodone, morphine, codeine, isoflurane, sevoflurane, acetaminophen, exemestane |
| Management of opioid addiction | *UGT2B7* | methadone, fentanyl |
| **Viral and microbial infection** | | |
| Antivirals | *CYP2A6, SLC22A2, CYP2R1, GSTM1* | efavirenz , lamivudine, zidovudine, ribavirin, nevirapine |
| Antibiotics | *CYP2A6, SLCO2B1, UGT2B7, GSTM1, GSTP1, GSTT1* | sulfonamides, metronidazole, streptomycin, rifampin, isoniazid |
